# Delivery of different genes into pre- and post-synaptic neocortical interneurons connected by GABAergic synapses

**DOI:** 10.1371/journal.pone.0217094

**Published:** 2019-05-24

**Authors:** Aarti Nagayach, Anshuman Singh, Angel L. De Blas, Alfred I. Geller

**Affiliations:** 1 Department of Ophthalmology, Louisiana State University Health Sciences Center, New Orleans, Louisiana, United States of America; 2 Department of Physiology and Neurobiology, University of Connecticut, Storrs, Connecticut, United States of America; 3 Department of Pharmacology, Louisiana State University Health Sciences Center, New Orleans, Louisiana, United States of America; Nanjing University, CHINA

## Abstract

Local neocortical circuits play critical roles in information processing, including synaptic plasticity, circuit physiology, and learning, and GABAergic inhibitory interneurons have key roles in these circuits. Moreover, specific neurological disorders, including schizophrenia and autism, are associated with deficits in GABAergic transmission in these circuits. GABAergic synapses represent a small fraction of neocortical synapses, and are embedded in complex local circuits that contain many neuron and synapse types. Thus, it is challenging to study the physiological roles of GABAergic inhibitory interneurons and their synapses, and to develop treatments for the specific disorders caused by dysfunction at these GABAergic synapses. To these ends, we report a novel technology that can deliver different genes into pre- and post-synaptic neocortical interneurons connected by a GABAergic synapse: First, standard gene transfer into the presynaptic neurons delivers a synthetic peptide neurotransmitter, containing three domains, a dense core vesicle sorting domain, a GABA_A_ receptor-binding domain, a single-chain variable fragment anti-GABA_A_ ß2 or ß3, and the His tag. Second, upon release, this synthetic peptide neurotransmitter binds to GABA_A_ receptors on the postsynaptic neurons. Third, as the synthetic peptide neurotransmitter contains the His tag, antibody-mediated, targeted gene transfer using anti-His tag antibodies is selective for these neurons. We established this technology by expressing the synthetic peptide neurotransmitter in GABAergic neurons in the middle layers of postrhinal cortex, and the delivering the postsynaptic vector into connected GABAergic neurons in the upper neocortical layers. Targeted gene transfer was 61% specific for the connected neurons, but untargeted gene transfer was only 21% specific for these neurons. This technology may support studies on the roles of GABAergic inhibitory interneurons in circuit physiology and learning, and support gene therapy treatments for specific disorders associated with deficits at GABAergic synapses.

## Introduction

Neocortical GABAergic inhibitory interneurons play critical roles in synaptic plasticity, circuit physiology, and learning. Moreover, a number of neurological disorders are associated with defects in GABAergic transmission in the neocortex, including schizophrenia, autism, and other intellectual disabilities [1]. Of note, advanced cognitive tasks are encoded in distributed forebrain circuits that span multiple neocortical areas. Within a neocortical area, complex local circuits support information processing, and neurons are interconnected into functional columns [2, 3]. These local circuits contain tens to hundreds or thousands of different neuron types, and each type forms precise synaptic connections with other neuron types [4]. GABAergic neurons comprise ten to thirty percent of the neurons in a specific neocortical area, and GABAergic synapses represent only five to fifteen percent of neocortical synapses [5–7]. Thus, understanding local circuit information processing is challenging due to the size and complexity of these circuits. Genetic strategies for analyzing circuit physiology have tremendous potential for studying this problem, however, these strategies usually affect an entire circuit, or a large part of a circuit [8–11]. Thus, understanding local circuit physiology and information processing would benefit from a gene transfer technology that can selectively deliver different genes into the pre- and post-synaptic neurons that are located in specific neocortical layers, and connected by GABAergic synapses.

Enumerating GABAergic neuron types, physiological ensembles, and synapse types is an area of active research [5–7]. Initial studies used calcium binding proteins to define three subtypes of GABAergic neurons that contain parvalbumin, or calretinin, or calbindin. Modern transcriptomics analyses have revealed at least several tens of neocortical GABAergic neuron types, and specific GABAergic neuron types are found in specific neocortical layers. Further, transcriptomics coupled with neuroanatomical and physiological techniques suggest that specific GABAergic neuron types can be functionally grouped together based on a smaller number of repeated themes or motifs. Much work remains to be done to definitively enumerate GABAergic neuron types and physiological types. Nonetheless, understanding the roles of specific neocortical GABAergic neuron types in circuit physiology, learning, and specific disorders will require technology to selectively modify these neurons.

Established genetic technologies for mapping and/or modifying circuits focus on connections between areas, which in the forebrain are predominately glutamatergic, and these technologies lack the capability to deliver different genes into neurons connected by a specific synapse type. mGRASP can visualize the synapses that connect specific neuron types, but is not synapse type-specific, and gene transfer is not selective for the connected neurons [12, 13]. Retrograde or anterograde connections have been mapped using specific viruses, but these approaches deliver the same gene(s) into all the infected neurons [14]. A Rabies Virus-based technology can deliver a gene into postsynaptic neurons, and then deliver a different gene into all the presynaptic neurons [15], but is limited to a specific transgenic mouse, lacks synapse type specificity, and physiological experiments are limited to ~5–11 days after transduction as Rabies Virus is neurotoxic and lethal [15]. Moreover, these technologies have been applied predominately to connections between areas, and it may be problematic to use these technologies to study local circuits within a neocortical area.

For the model system to develop gene transfer selectively across local GABAergic synapses, we studied projections from GABAergic neurons in the middle layers of postrhinal (POR) cortex to GABAergic neurons in the upper layers of this area. These neurons and synapses are part of a large distributed neocortical circuit that can encode visual object learning [16, 17]. POR cortex receives inputs from a number of neocortical visual areas, and sends projections to more than ten neocortical areas and the hippocampus [18, 19]. We established a genetic intervention in POR cortex that enables the genetically-modified circuit to encode some of the essential information for visual object learning: Activation of protein kinase C (PKC) pathways in several hundred GABAergic and glutamatergic neurons (using a virus vector) increases activation-dependent neurotransmitter release and improves accuracy for new visual object discriminations. Interestingly, the local circuit in POR cortex is preferentially activated during this learning [10, 17, 20]. Further, this local circuit encodes essential information; after gene transfer and learning, lesioning the genetically-modified, local circuit reduces performance [17]. Of note, there are high levels of GABA_A_ ß2 and ß3 subunits in POR cortex (Allen Brain Atlas; http://mouse.brain-map.org), and these subunits are recognized by the antibody we used for targeting gene transfer across GABAergic synapses.

We previously established a strategy to deliver different genes into pre- and post-synaptic neurons in different neocortical areas that are connected by glutamatergic synapses [21], and here we adapted this technology to deliver different genes into pre- and post-synaptic local interneurons that are connected by GABAergic synapses. The presynaptic vector expresses a synthetic peptide neurotransmitter that contains a single-chain variable fragment (ScFv) anti-GABA_A_ ß2 and ß3, and the His tag. This peptide was expressed in GABAergic neurons in the middle layers of POR cortex by using a GABAergic-specific promoter. Upon release, this synthetic peptide neurotransmitter can bind to GABA_A_ receptors on postsynaptic neurons in the upper layers of POR cortex. Antibody-mediated, targeted gene transfer then delivers a gene selectively into these postsynaptic neurons, using an anti-His tag antibody that binds to the His tag in the synthetic peptide neurotransmitter. This technology will have multiple applications to both basic neuroscience and gene therapy. This technology may enable studies on the role of inhibitory interneurons, and local neocortical circuits, in synaptic plasticity, circuit physiology, and learning. Local circuits could be mapped in detail, using this technology. Further, this technology may benefit developing gene therapies for specific disorders in which GABAergic neurons play a critical role.

## Materials and methods

### Ethics statement

All animal care and experimental procedures were approved by the Louisiana State University Health Science Center New Orleans Institutional Animal Care and Use Committee and followed the National Institutes of Health Guide for the Care and Use of Laboratory Animals.

### ScFv anti-GABA_A_ ß2 or ß3

The mouse monoclonal anti-GABA_A_ ß2 or ß3 antibody 62-3G1 [22, 23] recognizes an extracellular epitope on the ß2 and ß3 subunits [24]. The DNA sequences for both the heavy and light chains were commercially determined by cDNA cloning and sequencing (S1 and S2 Figs; MCLAB).

Blast searches (ncbi.nlm.nih.gov/igblast/igblast.cgi) revealed that the light chain sequence is closest to the IGKV1-135*01 and the IGKJ1*01 germ line genes, and the heavy chain sequence is closest to the IGHV1-82*01, IGHD4-1*02, and IGHJH2*01 germ line genes. A long open reading frame is present in only one of the six frames (including the complementary strand) for either the light or heavy chains, and the corresponding aa sequences were obtained (MacVector). The program abYsis (abysis.org) recognized these aa sequences as either a light chain or heavy chain variable region. These DNA sequences were used to construct ScFv, which were inserted into synthetic peptide neurotransmitters, as detailed below with DNA sequence.

For constructing two ScFv, the two variable regions were joined by a standard linker, (Gly_4_Ser)_3_ [25]; a DNA sequence for this linker was obtained by reverse translation with rat codon biases (MacVector). Either the light or heavy chain sequence was placed at the 5’ end, the linker was inserted, followed by the sequence for the other variable chain.

### Vectors

To obtain GABAergic-specific expression of synthetic peptide neurotransmitters, we used a 10.1 kb mouse GAD67 promoter. We previously established that a Herpes Simplex Virus (HSV-1) vector containing this promoter supports 88% GABAergic-specific expression of ß-galactosidase in rat POR cortex [26]. As this vector does not contain convenient restriction sites for replacing the *Lac Z* gene with other genes, we constructed a suitable vector: A 1.7 kb GAD promoter fragment extending from a naturally occurring *Not I* site to just before the ATG, followed by artificial *Hind III* and *Asc I* sites, was commercially synthesized and inserted into pUC57 (Genscript Inc.). This plasmid was digested with *Not I* and *Asc I*, and the 1.7 kb GAD promoter fragment was inserted into pVGLUT1dcv-secretogranin/anti-NR1-10aa/his-tag [21] that had been digested with the same enzymes (which excise the VGLUT1 promoter), yielding pGAD1.7KBdcv-secretogranin/anti-NR1-10aa/his-tag. pmGAD67-lacZ [27] (gift from Dr. Y. Yanagawa), which contains a 10.2 kb fragment of the mouse GAD67 promoter, was digested with *Hind III*, and two oligonucleotides were inserted (sense 5’ AGCTTGTTTAAACTTATCGATAACGGAACCTCTGG 3’ and antisense 5’ AGCTCCAGAGGTTCCGTTATCGATAAGTTTAAACA 3’) to remove this site and add *Pme I* and *Cla I* sites, yielding pmGAD67-lacZΔH3+Pme-Cla. A *Not I* site was subsequently discovered in a polylinker proximal to the 5’ end of the GAD promoter; to remove this site, pmGAD67-lacZΔH3+Pme-Cla was partially digested with *Not I*, and two oligonucleotides (sense 5’ GGCCAAGGAACCCTTCTCAGAGTCAAGTT 3’ and antisense 5’ GGCCAACTTGACTCTGAGAAGGGTTCCTT 3’) were inserted into the linear fragment, yielding pmGAD67-lacZΔH3-Not+Pme-Cla. pmGAD67-lacZΔH3-Not+Pme-Cla was digested with *Cla I* and *Not I*, and the 8.3 kb GAD promoter fragment was inserted into pGAD1.7KBdcv-secretogranin/anti-NR1-10aa/his-tag that had been digested with the same enzymes, yielding pGADdcv-secretogranin/anti-NR1-10aa/his-tag. To enable routine insertion of additional transcription units for future physiological experiments, this vector was digested with *Cla I* and two oligonucleotides were inserted to add unique *AsiS I*, *Mlu I*, and *Pme I* sites at the 5’ end of the GAD promoter (sense 5’ CGATGCGATCGCAGAACGCGTCAGGTTTAAACGG 3’; antisense 5’ CGCCGTTTAAACCTGACGCGTTCTGCGATCGCAT 3’), yielding pGADdcv-secretogranin/anti-NR1-10aa/his-tag+amp.

Two vectors were constructed that express a synthetic peptide neurotransmitter which contains a ScFv anti-GABA_A_ ß2 or ß3. The synthetic peptide neurotransmitter dcv-secretogranin/anti-GABA_A_ß2or3-HtoL/his-tag was based on dcv-secretogranin/anti-NR1-10aa/his-tag, and dcv-pomc/anti-GABA_A_ß2or3-LtoH/his-tag was based on dcv-pomc/anti-NR1-var/his-tag [21]. A Kozak consensus translation initiation sequence was placed 5’ to the ATG for each synthetic peptide neurotransmitter [28, 29]. The human Secretogranin II dense core vesicle (DCV) sorting domain contains the signal peptide and amino acids (aa) 1–41 [30, 31], or the mouse pro-opiomelanocortin (POMC) DCV sorting domain contains the signal peptide and aa 1 to 101 [32, 33]. These DCV sorting domains have been established in synthetic peptide neurotransmitters [21]. To reduce any steric hindrance between either DCV sorting domain and either ScFv, we inserted a 42 bp spacer between these domains; this spacer was derived from a linker used in ScFv (GSTSGSGKSSEGKG) [25]; a DNA sequence was obtained using reverse translation with rat codon biases (MacVector). This spacer was followed by a ScFv, a previously established 60 bp spacer for separating a receptor binding domain from the His tag [21], and the His tag. These two synthetic peptide neurotransmitters (dcv-secretogranin/anti-GABA_A_ß2or3-HtoL/his-tag, dcv-pomc/anti-GABA_A_ß2or3-LtoH/his-tag) were commercially synthesized (Genscript Inc.) and inserted into pUC57. S3 and S4 Figs contain the DNA sequences. The two plasmids containing these synthetic peptide neurotransmitters were digested with *Asc I* and *Pac I*, and the fragments containing the synthetic peptide neurotransmitters were inserted into the large fragment from pGADdcv-secretogranin/anti-NR1-10aa/his-tag+amp that had been digested with the same enzymes, yielding pGADdcv-secretogranin/anti-GABA_A_ß2or3-HtoL/his-tag or pGADdcv-pomc/anti-GABA_A_ß2or3-LtoH/his-tag.

We have previously described the two postsynaptic vectors, pINS-TH-NFHdendrite-gfp [34] and pINS-TH-NFHdendrite-PkcΔGG [21]. Two dendrite-targeting sequences, a myristoylation/palmitoylation site, from *Fyn*, and a basolateral (dendrite) membrane-sorting domain, from the low density lipoprotein receptor, were fused to GFP, yielding dendrite-targeted GFP [35]. To construct dendrite-targeted PkcΔGG, the GFP domain in dendrite-targeted GFP was replaced with a mutated rat protein kinase C (PKC) ßII catalytic domain fused to the flag tag; this point mutation abolishes PKC enzyme activity [36].

These constructs are available to academic researchers by written request to the corresponding author.

### Cells and vector packaging

2–2 cells, used for HSV-1 vector packaging, and BHK21 cells, used for titering, were grown in Dulbecco’s Modified Minimal Essential Medium (DMEM) with 10% FBS, 4 mM glutamine, and penicillin/streptomycin. Cultures were maintained at 37 °C in the presence of 5% CO_2_, and 100% humidity. 2–2 cells express the HSV-1 immediate early 2 (IE 2) gene [37]; these cells were grown in the presence of G418 (0.5 mg/ml), which was removed before the cells were plated for packaging. Vector stocks were titered using confluent (late-log phase) cultures of BHK21 cells. Both presynaptic vectors and the control postsynaptic vector (pINS-TH-NFHdendrite-PkcΔGG) were packaged using the standard procedure [38, 39]. To support gene transfer to connected neurons, the experimental postsynaptic vector (pINS-TH-NFHdendrite-gfp) was packaged for antibody-mediated, targeted gene transfer, using gC—ZZ (a chimeric glycoprotein C (gC) that contains an immunoglobulin G (IgG) binding domain, the *Staphylococcus* A protein ZZ domain and gBpK^-^, a mutated gB that lacks the glycosaminoglycan binding domain that binds to many cell types) [21, 40]. Two mouse anti-His tag antibodies (a 1:1 mixture of Qiagen 34650 and 34660, RRID:AB_2714179 and RRID:AB_2687898; 5 mg/ml total antibody during the vector binding procedure) were incubated with these vector particles (Zhang et al., 2012b). The titers for the resulting purified vector stocks (infectious vector particles per ml (IVP/ml)) were determined using BHK cells; 24 h after transduction, cells were fixed and immunocytochemistry was performed. For vector stocks that support antibody-mediated, targeted gene transfer, we have established that the titers of vector genomes (VG/ml), and the packaging efficiencies (VG/ml / IVP/ml), are similar to other vector stocks prepared using standard packaging [26, 40–44]. Thus, we did not determine VG/ml on the vector stocks used in this study. The titers of the vector stocks used here are: pGADdcv-secretogranin/anti-GABA_A_ß2or3-HtoL/his-tag 1.5×10^6^ IVP/ml, pGADdcv-pomc/anti-GABA_A_ß2or3-LtoH/his-tag 2.1×10^6^ IVP/ml, pINS-TH-NFHdendrite-gfp/gC–ZZ+anti-His tag 2.8×10^6^ IVP/ml, and pINS-TH-NFHdendrite-PkcΔGG 2.2×10^6^ IVP/ml. Wild-type HSV-1 was not detected (<10 plaque forming units/ml) in any of the vector stocks used here.

### Gene transfer

Long-Evans rats (male, 200–220 g) were purchased from Charles River. Water and food were available *ad libitum*, and the rats were maintained on a 12h/12h light-dark cycle.

Stereotactic surgeries were performed using our previously established procedures [21, 44]. Briefly, rats were anesthetized using a mixture of Ketamine and Xylazine (20 mg/ml or 2 mg/ml; final concentrations of 60 mg/kg or 6 mg/kg), and placed in a small rodent stereotactic frame; additional anesthesia was administered on an as needed basis. For the presynaptic gene transfer, a single injection was delivered into the middle layers of POR cortex (left hemisphere; anterior-posterior (AP) -8.0, medial-lateral (ML) -6.0, dorsal-ventral (DV) -5.2 [21, 45]). For the postsynaptic gene transfer, a 1:1 (IVP:IVP) mixture of the experimental and control postsynaptic vectors was delivered by a single injection into the upper layers of POR cortex (left hemisphere; AP -7.68, ML -6.8, DV -6.0). AP was measured from bregma, ML from the sagittal suture, and DV from the bregma-lambda plane. The dimension of POR cortex from the neocortical surface to the white matter is at angles relative to both the plane of the midline and the horizontal plane. Thus, these injection coordinates were designed to deliver the pre- and post-synaptic vectors into the same neuronal columns in POR cortex. Injections were performed using a micropump (KD Scientific model 100); 3 μl of vector stock was slowing injected over 5 minutes, and after an additional 5 minutes, the needle was slowly retracted.

### Immunohistochemistry

Following perfusions [44], 25 μm free-floating coronal sections that contained POR cortex were prepared using a freezing microtome. These sections were used for immunofluorescent assays, using our established protocol [44]. For assaying gene transfer to connected neurons, the primary antibodies were rabbit anti-His tag (Cell Signaling RRID:AB_10691695, 1:200 dilution) and either mouse anti-GFP (ThermoFisher RRID:AB_2334927, 1:500 dilution) or mouse anti-flag (Sigma RRID:AB_259529, 1:500 dilution). These antibodies were visualized using Texas red-conjugated goat anti-rabbit IgG (Vector RRID:AB_2336199, 1:200 dilution) and FITC-conjugated goat anti-mouse IgG (ThermoFisher RRID:AB_2534753, 1:200 dilution). The negative control was assays lacking the primary antibodies. For identifying the postsynaptic GABAergic neuron types, the primary antibodies were mouse anti-Parvalbumin (Sigma RRID:AB_477329, 1:800 dilution), or mouse anti-Calretinin D29K (Synaptic Systems, RRID:AB_2619904, 1:1,000 dilution), or mouse anti-Calbindin D-28k (Sigma RRID:AB_476894, 1:50 dilution); and these assays also contained rabbit anti-His tag (Cell Signaling RRID:AB_2744546, 1:5,000 dilution) and rat anti-GFP (Santa Cruz RRID:AB_1124404, 1:4,000 dilution). These primary antibodies were visualized using TRITC-conjugated goat anti-mouse IgG (Sigma RRID:AB_261768, 1:500 dilution), fluorescein-conjugated goat anti-rabbit IgG (Vector RRID:AB_2336197, 1:500 dilution), and Alexa Fluor 633-conjugated goat anti-rat IgG (ThermoFisher RRID:AB_2535749, 1:500 dilution). The dendrite-targeted GFP does support autofluorescence of GFP, but a much stronger signal is obtained by amplification with primary and secondary antibodies.

A Leica TCS SP8 X confocal microscope was used to obtain photomicrographs. Multiple images of the same field were obtained using microscope settings designed to detect the indicated secondary antibodies. For each field that was analyzed for gene transfer to connected neurons, a single image was obtained for each channel, thereby ensuring that processes proximal in the X and Y dimensions were also proximal in the Z dimension. We did not examine “stacks” of images in the Z dimension. Gene transfer to connected neurons was quantified by merging the two images that contained His tag-immunoreactivity (IR) and either GFP-IR or flag-IR using Adobe Photoshop; the connected axons and dendrites were scored in the merged images. In the fields that were examined, all the His tag-IR axons were scored for being proximal to, or distant from, either a GFP-IR or a flag-IR dendrite. To quantify the specific postsynaptic GABAergic types, synapses for the connected neurons were identified as just described; and the image for a specific neuron type maker was then merged with the image of synapses for the transduced connected neurons. The previously marked synapses were scored for containing, or lacking, the indicated GABAergic type marker.

### Statistical analyses

StatPlus was used to perform descriptive statistical analyses and one-way ANOVAs followed by Neuman-Keuls posthoc test.

## Results

### The strategy for delivering different genes into pre- and post-synaptic local interneurons connected by GABAergic synapses

**Fig 1:**
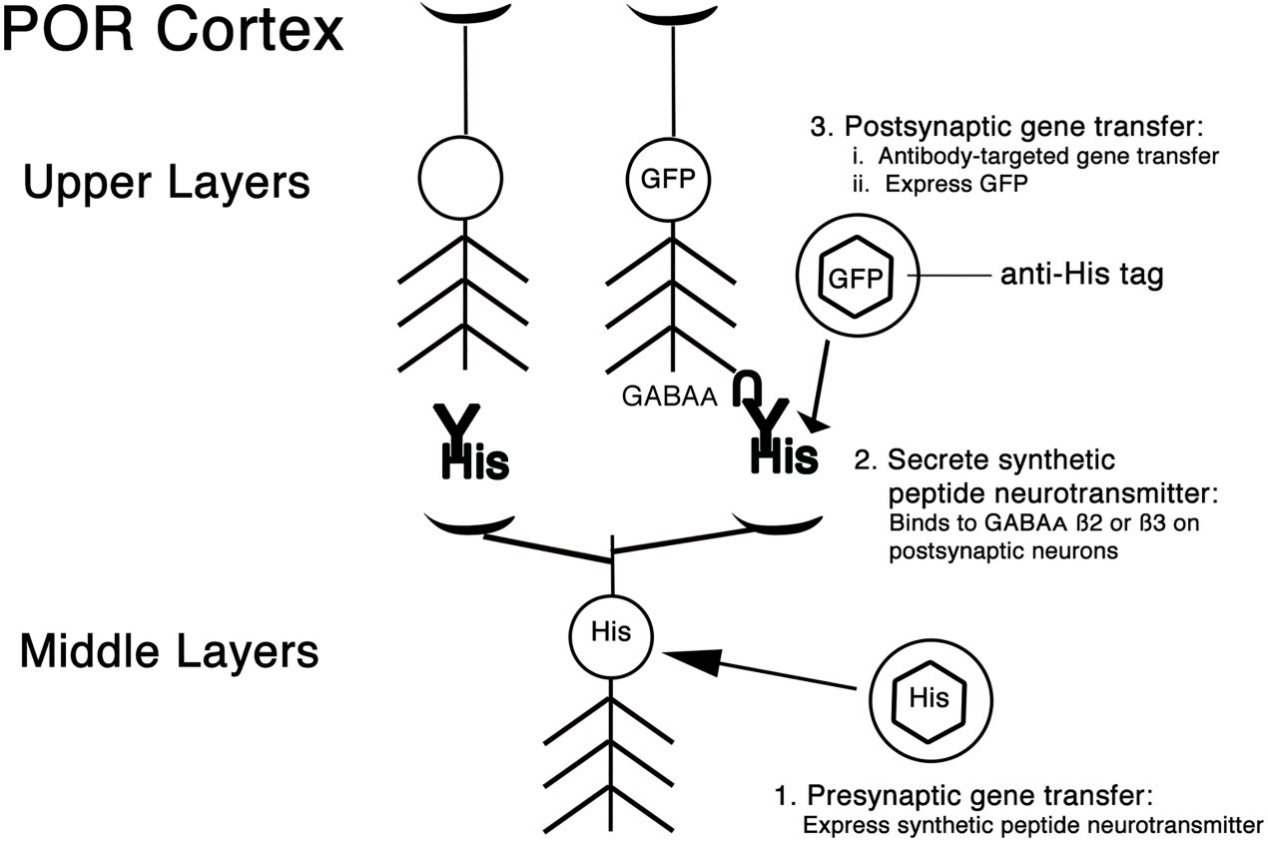
The strategy for delivering different genes into pre- or post-synaptic neurons in POR cortex connected by GABAergic synapses that contain GABA_A_ ß2 or ß3 subunits. This strategy contains three steps: Step 1 is the presynaptic gene transfer: This gene transfer uses an injection site in the middle layers of POR cortex, and the gene transfer uses standard procedures. This vector expresses a synthetic peptide neurotransmitter from a GABAergic-specific promoter, the GAD67 promoter. The synthetic peptide neurotransmitter, abbreviated His within a HSV-1 vector particle (hexagon surrounded by a circle), contains three domains: i) a DCV sorting domain, ii) a ScFv anti-GABA_A_ ß2 or ß3, and iii) the His tag. Step 2 is release of the synthetic peptide neurotransmitter (shown as His-Y, Y indicates the ScFv domain), followed by binding to GABA_A_ receptors (shown as inverted U) on the postsynaptic neuron on the right side of the Fig. Step 3 is the postsynaptic gene transfer: This gene transfer uses an injection site is in the upper layers of POR cortex and is selective for the postsynaptic neurons that have bound the synthetic peptide neurotransmitter: The gene transfer uses antibody-mediated targeting. These vector particles contain a modified HSV-1 glycoprotein fused to the IgG binding domain from *Staphylococcus* A protein, and bound to anti-His tag antibody (in the top right of the Figure: HSV-1 vector particle, hexagon surrounded by a circle; modified HSV-1 glycoprotein, horizontal line from HSV-1 vector particle to anti-His tag). Complexes of these vector particles and anti-His tag antibodies are injected, and these complexes bind to the His tag on the synthetic peptide neurotransmitter, bound to GABA_A_ ß2 or ß3 subunits on specific postsynaptic neurons. To support assays for gene transfer to connected neurons, the postsynaptic vector expresses a dendrite-targeted GFP from a pan-neuronal promoter, the INS-TH-NFH promoter. This system supports highly specific gene transfer to neurons connected by GABA_A_ ß2 or ß3 subunit-containing synapses: As shown in the left column, axon collaterals from transduced neurons will release the synthetic peptide neurotransmitter at other synapses, but if the postsynaptic neuron lacks GABA_A_ ß2 or ß3 subunits, that postsynaptic neuron will not be transduced.

The strategy contains three steps (Fig 1). Step 1 is gene transfer into the presynaptic neurons. This step uses standard gene transfer procedures, and these vector particles were injected into the middle layers POR cortex. This vector expresses a synthetic peptide neurotransmitter that has three domains. To enable processing and release of the recombinant protein as a peptide neurotransmitter, the N-terminal domain is a DCV sorting domain [46]. The middle domain binds to a specific receptor on the postsynaptic neurons. Our first study reported gene transfer across glutamatergic synapses by using sequences from an anti-NMDA NR1 subunit antibody, as NR1 is found in most glutamatergic synapses [21, 47, 48]. More recently, to target gene transfer across *TrkB*-containing synapses, we used BDNF; or to target across NR2B- or mGluR5-containing synapses, we used ScFv anti-NR2B or ScFv anti-mGluR5. *TrkB*, NR2B, and mGluR5 are present in specific subsets of glutamatergic synapses. Here, to target across GABAergic synapses, we used ScFv anti-GABA_A_ ß2 or ß3. The C-terminal domain of the synthetic peptide neurotransmitter is the His tag, which supports antibody-mediated, targeted gene transfer. Step 2 is that following release from the presynaptic neurons, the synthetic peptide neurotransmitter selectively binds only to postsynaptic neurons that express its cognate receptor. Step 3 is gene transfer selectively into postsynaptic neurons that form synapses with the transduced presynaptic neurons. This gene transfer uses antibody-mediated, targeted gene transfer [40, 41] and an anti-His tag antibody, as the synthetic peptide neurotransmitter contains the His tag. For this purpose, an IgG binding domain, the *Staphylococcus* A protein ZZ domain, was fused to a HSV-1 encoded glycoprotein (gC) on the surface of the vector particles (gC—ZZ chimeric protein), and anti-His tag antibody is bound to these vector particles. This postsynaptic gene transfer is into the upper layers of POR cortex. Thus, gene transfer to connected neurons is between local GABAergic neurons in the middle and upper layers of POR cortex.

This strategy confers significant specificity for both neuron- and synapse-type (Fig 1). The postsynaptic gene transfer is limited to neurons that both receive a synapse from a transduced presynaptic neuron and express the cognate receptor for the synthetic peptide neurotransmitter. Proximal postsynaptic neurons that receive a synapse from the same transduced presynaptic neuron, via an axon collateral, but do not express the indicated receptor, are not transduced. More generally, postsynaptic neurons that receive a synapse from a transduced presynaptic neuron, but do not express the indicated receptor, are not transduced. Further, other proximal postsynaptic neurons that express the indicated receptor, but do not receive a synapse from a transduced presynaptic neuron, are not transduced. Moreover, although the presynaptic neurons may project to multiple neocortical layers and neurons, only postsynaptic neurons proximal to the second injection site, and the postsynaptic vector particles, are transduced. Of note, additional specificity for neuron-type is available by appropriate choice of promoter for both the pre- and the post-synaptic vectors. Thus, in the forebrain, a specific presynaptic interneuron forms hundreds to thousands of synapses, but this strategy restricts the postsynaptic gene transfer to a limited subset of the postsynaptic neurons, specifically, those located in a specific layer or location and connected by a specific synapse type. Thus, this strategy has advantages in specificity compared to the other technologies for gene transfer to connected neurons that are detailed in the introduction (Table 1).

**Table 1:** Comparison of the different technologies for delivering genes into connected neurons.

Technology	Specific for connected neurons^a^	Deliver different genes into connected neurons	Specific for connected neurons by brain area/location^b^	Synapse type-specific	Neurotoxic
mGRASP using a virus vector^c^	no	yes	yes	no	no
Circuit mapping using viruses^d^	yes	no	no	no	yes
Rabies virus-based gene transfer to connected neurons^e^	yes	yes	no	no	yes
Synthetic peptide neurotransmitters^f^	yes	yes	yes	yes	no

^a^Following the first gene transfer, is subsequent gene transfer limited to connected neurons?

^b^Following the first gene transfer, does gene transfer into the connected neurons deliver the second gene into all the connected neurons regardless of brain area or location, or can the second gene transfer be limited to connected neurons in a specific brain area or location?

^c^[12, 13]

^d^[14]

^e^[15]

^f^[21]

### Synthetic peptide neurotransmitters containing ScFv anti-GABA_A_ ß2 or ß3, and the vectors for gene transfer across GABAergic synapses

Using cDNA cloning and sequencing, a commercial vendor (MCLAB) determined the DNA sequences for the light and heavy chains for a mouse monoclonal anti-GABA_A_ ß2 or ß3 antibody (62-3G1; [22, 23]). S1 and S2 Figs present these DNA sequences. This antibody recognizes an extracellular epitope in the GABA_A_ receptor ß2 and ß3 subunits [24]. Of note, the ß2 and ß3 subunits are widely expressed in the forebrain (Allen Brain Atlas, mouse.brain-map.org). Thus, ScFv based on this antibody are appropriate to use in synthetic peptide neurotransmitters designed to bind to GABA_A_ receptors, and these synthetic peptide neurotransmitters should be capable of targeting gene transfer across many GABAergic synapses in the forebrain. To design ScFv, either the light chain or heavy chain variable region sequence was placed at the 5’ end, a linker commonly used to connect the chains in ScFv was inserted ((Gly_4_Ser)_3_ [25]), followed by the sequence for the other variable chain.

**Fig 2:**
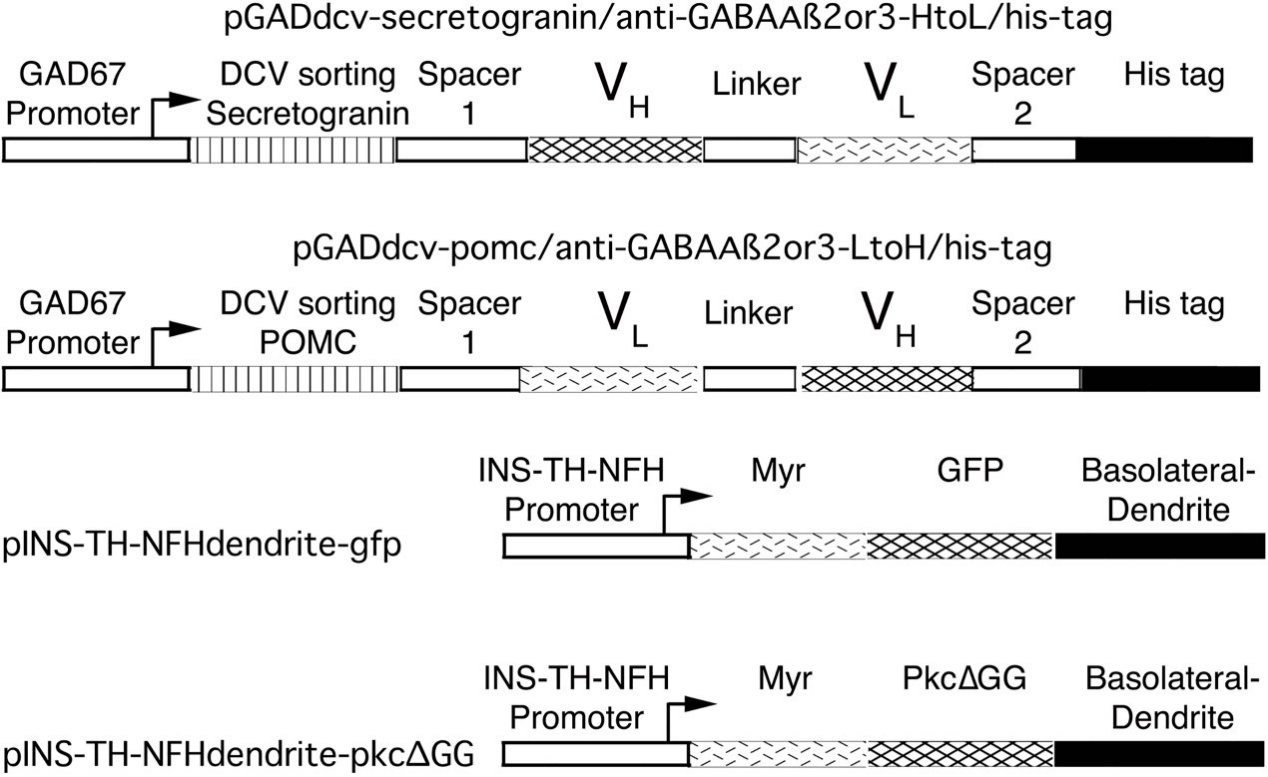
The transcription units in the pre- or post-synaptic vectors. These four vectors all contain a standard HSV-1 vector backbone [21]. The two presynaptic vectors use the GABAergic-specific GAD67 promoter to express a synthetic peptide neurotransmitter designed to bind to GABA_A_ ß2 or ß3 subunits. Each synthetic peptide neurotransmitter contains a DCV sorting domain, either from Secretogranin II or POMC, a spacer (spacer 1, 42 bp), a ScFv anti-GABA_A_ ß2 or ß3, another spacer (spacer 2, 60 bp), and the His tag. The ScFv contain either the heavy or light chain variable region, a established linker, (Gly_4_Ser)_3_ [25], and the other variable region. The two spacers separate the ScFv from the other domains, and are designed to enable each domain to function without steric hindrance from the other domains. The two postsynaptic vectors have been reported [21], and express a dendrite-targeted marker, either GFP or PkcΔGG (an enzymatically inactive PKC) from the pan-neuronal INS-TH-NFH promoter. Two dendrite-targeting signals are fused to each marker; first, a myristoylation/palmitoylation (Myr) site (from *Fyn)*, and, second, a basolateral/ dendrite membrane-sorting domain (from the low density lipoprotein receptor) [35].

Two synthetic peptide neurotransmitters containing ScFv anti-GABA_A_ ß2 or ß3 were derived by modifying established synthetic peptide neurotransmitters that support gene transfer across most glutamatergic synapses, and which were designed to recognize NMDA NR1 subunits [21]: We replaced the NR1 binding domains with a ScFv anti-GABA_A_ ß2 or ß3. To summarize, these new synthetic peptide neurotransmitters contain three domains (Fig 2; DNA sequences in S3 and S4 Figs): To enable the synthetic peptide neurotransmitter to be processed as a peptide neurotransmitter, the N-terminal domain is a DCV sorting domain from either human Secretogranin II [30, 31] or mouse POMC [32, 33]. The middle domain is a ScFv anti-GABA_A_ ß2 or ß3. The C-terminal domain is the His tag. To reduce any potential steric hindrance between the domains, specific spacers were inserted between the ScFv and the other domains.

Previous studies established that these DCV sorting domains support transport into axons, in glutamatergic neurons. POR cortex and perirhinal (PER) cortex have large, reciprocal connections [49]. After injection of vectors into POR cortex, there is minimal retrograde transport of vectors to cell bodies in PER cortex; POR cortex contained several hundred transduced neurons, and PER cortex contained less than five transduced neurons [10]. Also, after injection of vectors into POR cortex, the transduced processes in PER cortex are axons; an axon-targeted ß-galactosidase colocalized with the axon marker tau [21]. Additionally, after injection of vectors that express synthetic peptide neurotransmitters into POR cortex, transduced processes in PER cortex display axonal morphology [21].

As the present synthetic peptide neurotransmitters are designed to bind to specific GABA_A_ receptors, each synthetic peptide neurotransmitter was expressed from a GABAergic-specific promoter, the GAD67 promoter [26] (Fig 2). A previous study showed that this GAD promoter supports ~90% GABAergic-specific expression in POR cortex, from HSV-1 vectors, by costaining for a recombinant protein and GAD67 [26]. These presynaptic vectors were packaged into HSV-1 particles using standard helper virus-free packaging [38].

For marking the cell bodies and dendrites of the transduced postsynaptic neurons, we used a previously reported vector that expresses a dendrite-targeted GFP (Fig 2) [21]. This construct contains two sequences for dendrite-targeting, a myristoylation/palmitoylation site and a basolateral (dendrite) membrane-sorting domain [35]. Dendrite-targeted GFP was expressed from an established neuron-specific promoter; this promoter contains an insulator (INS), an upstream enhancer from the tyrosine hydroxylase (TH) promoter, and a neurofilament heavy gene (NFH) promoter [44]. The INS-TH-NFH promoter supports ≥90% neuron-specific expression in multiple forebrain areas, including specific neocortical areas. A previous study showed that a HSV-1 vector expressing this dendrite-targeted GFP labels cell bodies and dendrites; after injection into PER cortex, GFP colocalized with the dendrite marker MAP2 [21].

We packaged this vector (pINS-TH-NFHdendrite-gfp) for antibody-mediated, targeted gene transfer to neurons that contain the His tag on their surface [21]. To support antibody-mediated gene transfer, an IgG binding domain, the *Staphylococcus A* protein ZZ domain, was fused to HSV-1 gC, present on the surface of vector particles [40]. Following packaging, the resulting vector particles were incubated with anti-His tag antibody; these vector particle-antibody complexes were purified, and then used for gene transfer. Thus, these vector particle-antibody complexes will bind to the His tag in a synthetic peptide neurotransmitter, bound to its cognate receptor on the surface of a postsynaptic neuron. Subsequent entry of these vector particles into neurons uses the same mechanisms that support entry for wt HSV-1 [50].

The comparison, untargeted postsynaptic vector expresses a different marker that is also dendrite-targeted. The GFP domain in dendrite-targeted GFP was replaced with PkcΔGG (Fig 2). PkcΔGG is an enzymatically inactive, catalytic domain from rat PKC ßII; PkcΔGG contains a point mutation that blocks activity [21, 36]. This vector was packaged using the standard helper virus-free packaging procedure, with wt gC [38].

### Delivery of different genes into presynaptic and postsynaptic neurons connected by a GABAergic synapse that contains GABA_A_ ß2 or ß3 subunits

For the experimental design, the presynaptic vector was delivered into the middle layers POR cortex by stereotactic injection (one injection site, one hemisphere). Eight days later, a 1:1 mixture of the targeted and comparison (control) postsynaptic vectors was injected into the upper layers of POR cortex; the injection coordinates were designed to target the same neuronal columns as the presynaptic injection site. Four days later, the rats were sacrificed, and gene transfer to connected neurons was assayed. The eight-day period between the first and second gene transfers is intended to support presynaptic gene transfer, expression of the synthetic peptide neurotransmitter, followed by processing, transport, release, and binding to GABA_A_ receptors containing ß2 or ß3 subunits on the postsynaptic neurons. The four-day period between the second gene transfer and sacrificing the rats supports postsynaptic gene transfer, expression of the marker, and transport into dendrites. The timing of the two gene transfers, and rat sacrificing, followed an established design that supports gene transfer to neurons in POR and PER cortices, connected via glutamatergic synapses [21]. Delivering a mixture of the two postsynaptic vectors is designed to control for the inherent variation in injections sites between rats; in each rat, adjacent sections were analyzed for targeted (GFP) or untargeted, control (PkcΔGG) postsynaptic gene transfer.

**Fig 3:**
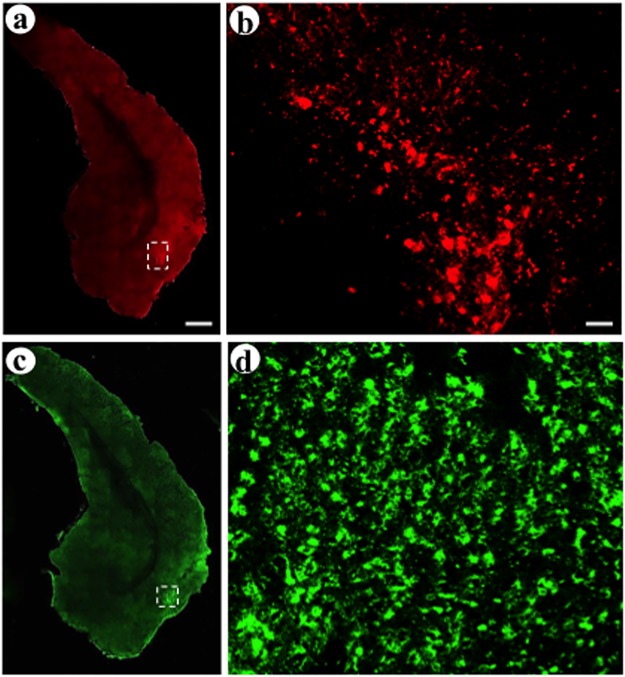
The locations of the pre- and postsynaptic injection sites in the middle or upper layers of POR cortex, and transduced neurons proximal to each injection site. (A and B) The presynaptic vector pGADdcv-secretogranin/anti-GABA_A_ß2or3-HtoL/his-tag, His tag-IR (Texas red-conjugated secondary antibody); (A) low power view, and (B) high power view of the box in panel A showing transduced neurons. (C and D) The postsynaptic vector pINS-TH-NFHdendrite-gfp/gC–ZZ+anti-His tag, GFP-IR (FITC-conjugated secondary antibody); (C) low power view, and (D) high power view of the box in panel C showing transduced neurons. Scale bars: (A and C) 1,200 μm; (B and D) 50 μm.

**Fig 4:**
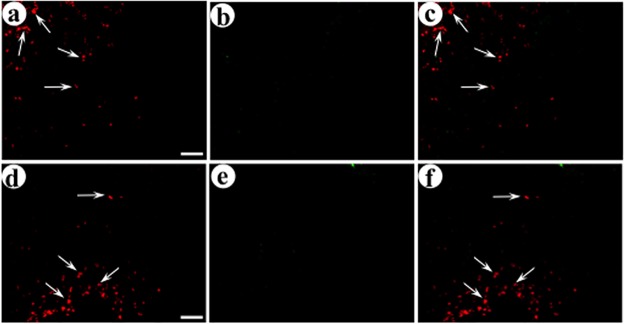
Recombinant proteins in the middle layers of POR cortex after delivery of the presynaptic vector, pGADdcv-secretogranin/anti-GABA_A_ß2or3-HtoL/his-tag, into the middle layers of POR cortex, followed by coinjection of the two postsynaptic vectors into the upper layers POR cortex. The presynaptic vector was injected, eight days later a 1:1 mixture of the two postsynaptic vectors was injected, and the rats were sacrificed four days later. The area proximal to the first, presynaptic gene transfer, in the middle layers of POR cortex, was analyzed for recombinant proteins by immunofluorescent costaining. Alternating sections were examined for the expression from the presynaptic vector and either the targeted or control postsynaptic vector. (A-C) Expression from pGADdcv-secretogranin/anti-GABA_A_ß2or3-HtoL/his-tag and the targeted postsynaptic vector, pINS-TH-NFHdendrite-gfp/gC–ZZ+anti-His tag; (A) His tag-IR (Texas red-conjugated secondary antibody), (B) GFP-IR (FITC-conjugated secondary antibody), and (C) merge. Arrows, neuronal cell bodies that express the synthetic peptide neurotransmitter from the presynaptic vector. (D-F) Costaining for expression from this presynaptic vector and the untargeted postsynaptic vector pINS-TH-NFHdendrite-PkcΔGG; (D) His tag-IR, (E) flag-IR (the flag tag is fused to PkcΔGG; FITC-conjugated secondary antibody), and (F) merge. Scale bar: 50 μm.

**Fig 5:**
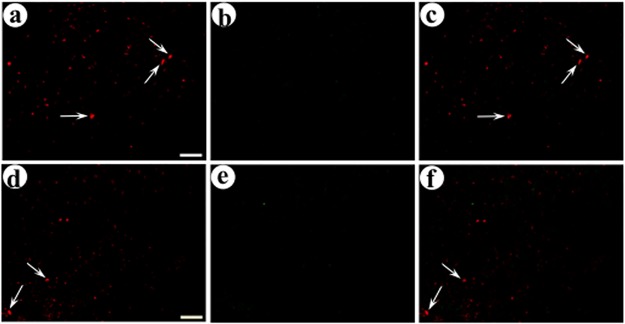
Recombinant proteins in the middle layers of POR cortex after injection of a presynaptic vector, pGADdcv-pomc/anti-GABA_A_ß2or3-LtoH/his-tag, into the middle layers of POR cortex followed by coinjection of the two postsynaptic vectors into the upper layers of POR cortex. The experimental design and immunofluorescent costaining were as above. (A-C) Expression from this presynaptic vector and the targeted postsynaptic vector; (A) His tag-IR, (B) GFP-IR, and (C) merge. Arrows, cell bodies expressing the synthetic peptide neurotransmitter from the presynaptic vector. (D-F) Costaining for this presynaptic vector and the control, untargeted postsynaptic vector; (D) His tag-IR, (E) flag-IR, and (F) merge. Scale bar: 50 μm.

We assayed expression of each synthetic peptide neurotransmitter in neurons with cell bodies in the middle layers of POR cortex, and we confirmed that the middle layers of POR cortex contained minimal expression from the postsynaptic vectors, as they were injected into the upper layers of POR cortex. HSV-1 particles can be transported retrogradely in axons, and significant levels of retrograde transport could complicate interpretation of the present data. However, we have observed only minimal levels of retrograde transport of these vectors between neocortical areas or between other brain areas [10, 51, 52]. Recombinant proteins expressed from each vector were detected using immunofluorescent assays, and a confocal microscope was used to obtain photomicrographs. Low power views show the positions of the pre- or post-synaptic injection sites in the middle or upper layers of POR cortex, and higher power views showed transduced neurons proximal to each injection site (Fig 3). Because neuronal columns in POR cortex are oriented at an angle relative to the coronal plane, different sections contain the pre- or post-synaptic injection sites. Proximal to the presynaptic injection site, in the middle layers of POR cortex, we observed neuronal-like cell bodies that supported expression of each synthetic peptide neurotransmitter (Fig 4, pGADdcv-secretogranin/anti-GABA_A_ß2or3-HtoL/his-tag; Fig 5, pGADdcv-pomc/anti-GABA_A_ß2or3-LtoH/his-tag). For reasons detailed below, for each field, we obtained a single confocal image in each channel (we did not collect image stacks); thus, specific cell bodies are shown in different planes and display different diameters. We also observed smaller processes, which likely represent labeling of proximal axons, due to transport of a synthetic peptide neurotransmitter into axons. In contrast, we observed minimal expression from either postsynaptic vector, as the postsynaptic vectors were injected into the upper layers of POR cortex, and the present photomicrographs captured the middle layers of POR cortex.

**Fig 6:**
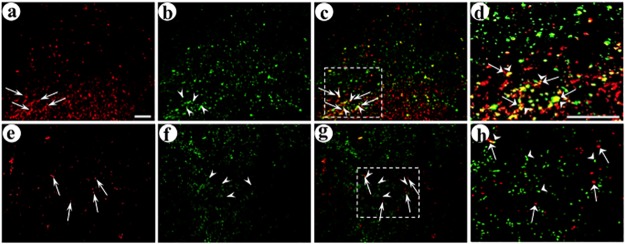
The synthetic peptide neurotransmitter in the presynaptic vector supports efficient gene transfer to connected neurons. The experimental design was as detailed in Fig 3 legend. Alternating sections containing POR cortex were analyzed for expression from the presynaptic vector and either the targeted or control postsynaptic vector, using immunofluorescent costaining. The upper layers of POR cortex were examined. (A-D) Delivery of this presynaptic vector, followed by the targeted postsynaptic vector, supports efficient gene transfer to connected neurons; (A) His tag-IR (Texas red-conjugated secondary antibody), (B) GFP-IR (FITC-conjugated secondary antibody), (C) merge, and (D) merge, an enlarged area showing specific transduced axons either proximal to, or distant from, a transduced dendrite. Arrows, transduced axons; arrowheads, transduced dendrites. (E-H) Delivery of this presynaptic vector followed by the untargeted, control postsynaptic vector supported only a low level of gene transfer to connected neurons; (E) His tag-IR, (F) flag-IR (FITC-conjugated secondary antibody), (G) merge, and (H) merge, an enlarged area with His tag-IR axons and flag-IR dendrites that are distant from each other. Scale bars: (A-C and E-G) 50 μm, and (D and H) 50 μm.

**Fig 7:**
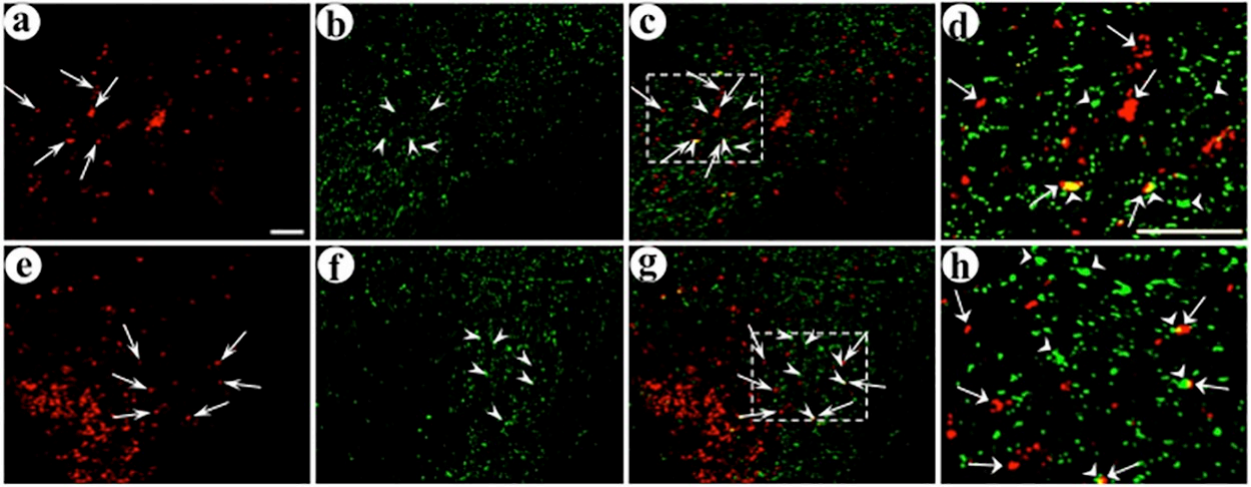
The synthetic peptide neurotransmitter dcv-pomc/anti-GABA_A_ß2or3-LtoH/his-tag supports limited gene transfer to connected neurons. The experimental design and immunofluorescent costaining were as above. (A-D) Delivery of this presynaptic vector followed by the targeted postsynaptic vector resulted in only low levels of gene transfer to connected neurons; (A) His tag-IR, (B) GFP-IR, (C) merge, and (D) merge, with an enlarged area that contains a number of transduced axons and dendrites; some transduced axons are proximal to a transduced dendrite and other transduced axons are distant from a transduced dendrite. Arrows, transduced axons; arrowheads, transduced dendrites. (E-H) Delivery of this presynaptic vector, followed by the untargeted postsynaptic vector, supported low levels of gene transfer to connected neurons, similar to those observed using the targeted postsynaptic vector; (E) His tag-IR, (F) flag-IR, (G) merge, and (H) merge, an enlarged area that shows transduced axons that are either proximal to, or distant from, transduced dendrites. Scale bars: (A-C and E-G) 50 μm, and (D and H) 50 μm.

We examined the area proximal to the postsynaptic injection sites, in the upper layers of POR cortex, for gene transfer to connected postsynaptic neurons. We assayed alternating sections for both axon terminals containing a synthetic peptide neurotransmitter and dendrites and cell bodies containing the marker expressed from either the targeted or control postsynaptic vector, specifically dendrite-targeted GFP or dendrite-targeted PkcΔGG. For each field, we obtained a single confocal image in each channel. A single confocal image has an ~1 μm depth of field; proximal axons and dendrites in the dimensions of width and height (X and Y dimensions) will also be proximal in depth (Z dimension). Using pGADdcv-secretogranin/anti-GABA_A_ß2or3-HtoL/his-tag and the targeted postsynaptic vector (expresses dendrite targeted-GFP), photomicrographs showed that many transduced axons were proximal to a transduced dendrite, and a limited number of the transduced axons were distant from a transduced dendrite (Fig 6A–6D). As detailed above, the DCV sorting domains used in these synthetic peptide neurotransmitters support transport into the axons of glutamatergic neurons; however, the present synthetic peptide neurotransmitters are expressed in GABAergic neurons (the presynaptic vectors contain a GAD67 promoter), and we observed these synthetic peptide neurotransmitters in many processes with axonal morphology. Many of these processes had a diameter consistent with axons, but some had a modestly larger diameter, which may be due to several technical issues, including diffraction of light through the 25 μm thick sections, fluorescence overexposure for axons with high levels of a synthetic peptide neurotransmitter, or image processing in Adobe Photoshop. We also detected the synthetic peptide neurotransmitter in a limited number of significantly thicker processes, which could represent retrograde transport of the vector from the injection site in the middle layers of POR cortex to cell bodies in the upper layers of POR cortex. The dendrite-targeted GFP was observed in both dendrites and cell bodies, as this protein is synthesized in cell bodies and then transported into dendrites. In contrast, for this same presynaptic vector and the comparison, untargeted postsynaptic vector (expresses dendrite targeted-PkcΔGG), photomicrographs showed that most transduced axons were distant from a transduced dendrite (Fig 6E–6H). However, for the other presynaptic vector, pVGLUT1dcv-pomc/anti-GABA_A_ß2or3-LtoH/his-tag, and the targeted postsynaptic vector, most of the transduced axons were distant from a transduced dendrite (Fig 7A–7D). Similarly, for this same presynaptic vector and the comparison postsynaptic vector, most transduced axons were distant from transduced dendrites (Fig 7E–7H). For each synthetic peptide neurotransmitter, and either the targeted or comparison (control) postsynaptic vector, we counted the numbers of transduced axons that were proximal to, or distant from, a transduced dendrite or cell body; an example of these counts is shown in S5A Fig, and S5A Fig legend details the procedure. These counts (Table 2) showed that dcv-secretogranin/anti-GABA_A_ß2or3-HtoL/his-tag supported a high, 61%, level of gene transfer to connected neurons, and for the comparison, untargeted postsynaptic vector, we observed a much lower level of gene transfer to connected neurons, only 21%. Of note, this synthetic peptide neurotransmitter supported significant gene transfer to connected neurons (targeted vs. untargeted postsynaptic vector, F_1,4_ = 31.9 p<0.005, 1-way ANOVA). However, the other synthetic peptide neurotransmitter, dcv-pomc/anti-GABA_A_ß2or3-LtoH/his-tag, did not support gene transfer to connected neurons; using the targeted postsynaptic vector, only 36% of the postsynaptic gene transfer was to connected neurons, and using the comparison postsynaptic vector, 30% of the postsynaptic gene transfer was to connected neurons (Table 2; targeted vs. untargeted postsynaptic vector F_1,4_ = 4.6 p>0.05). As pGADdcv-secretogranin/anti-GABA_A_ß2or3-HtoL/his-tag supported a high level of gene transfer to connected neurons, this vector was used in subsequent experiments.

**Table 2:** Targeted gene transfer across GABA_A_ ß2 or ß3-containing synapses; the percentage of transduced presynaptic axons proximal to a transduced postsynaptic dendrite.

Presynaptic vector	Postsynaptic vector			
	Targeting vector		Control vector	
	His-tag-IR axons	% GFP-IR costaining	His-tag-IR axons	% Flag-IR costaining
pGADdcv-secretogranin/anti-GABA_A_ß2or3-HtoL/his-tag	1,467±265	61±6	987±90	21±3
pGADdcv-pomc/anti-GABAAß2or3-LtoH/his-tag	1,106±86	36±2	723±58	30±2

In each section that was examined, all the transduced presynaptic axons were scored for being adjacent to, or distant from, a transduced postsynaptic dendrite. Three rats per experiment; three or four sections were scored for each condition; mean±s.e.m. are shown.

### Gene transfer across GABA_A_ ß2- or ß3-containing synapses delivers the postsynaptic vector into the three major subtypes of GABAergic neurons

**Fig 8:**
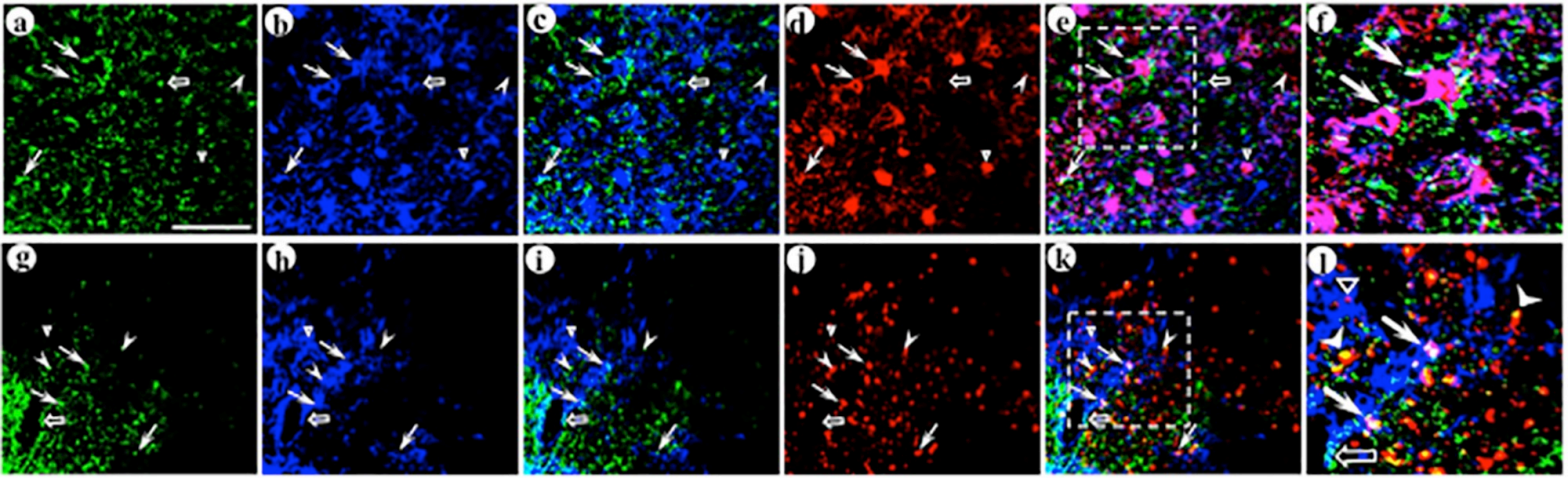
The synthetic peptide neurotransmitter dcv-secretogranin/anti-GABA_A_ß2or3-HtoL/his-tag supports gene transfer to the parvalbumin-containing subtype of GABAergic neurons. The experimental design was as above. Using immunofluorescent costaining, the upper layers of POR cortex were analyzed for expression from the presynaptic vector (His tag-IR), the targeted postsynaptic vector (GFP-IR), and the GABAergic subtype marker parvalbumin. (A-F) Image stacks show neuronal morphology for the transduced postsynaptic dendrites and cell bodies, proximal to transduced axons; (A) His tag-IR (fluorescein-conjugated secondary antibody), (B) GFP-IR (Alexa Fluor 633-conjugated secondary antibody), (C) merge of His tag-IR and GFP-IR, (D) parvalbumin-IR (TRITC-conjugated secondary antibody), (E) merge of all three IR, (F) merge, an enlarged area showing adjacent transduced axons and dendrites that contain parvalbumin-IR. Arrows, transduced axons proximal to transduced dendrites, with parvalbumin-IR. Open arrow, a transduced axon proximal to a transduced dendrite that lacks parvalbumin-IR. Arrowhead, a transduced axon distant from a transduced dendrite, but proximal to parvalbumin-IR. Open triangle, a transduced dendrite that contains parvalbumin-IR, but distant from a transduced axon. (G-L) Single confocal images in each channel show that delivery of this presynaptic vector, followed by the targeted postsynaptic vector, supported gene transfer across GABA_A_ ß2- or ß3-containing synapses to parvalbumin-containing postsynaptic neurons; (G) His tag-IR, (H) GFP-IR, (I) merge of His tag-IR and GFP-IR, (J) parvalbumin-IR, (K) merge of all three IR, (L) merge, an enlarged area showing adjacent transduced axons and dendrites that contain parvalbumin-IR. Panels G-L show a different field than for panels A-F. Scale bar: 20 μm.

**Fig 9:**
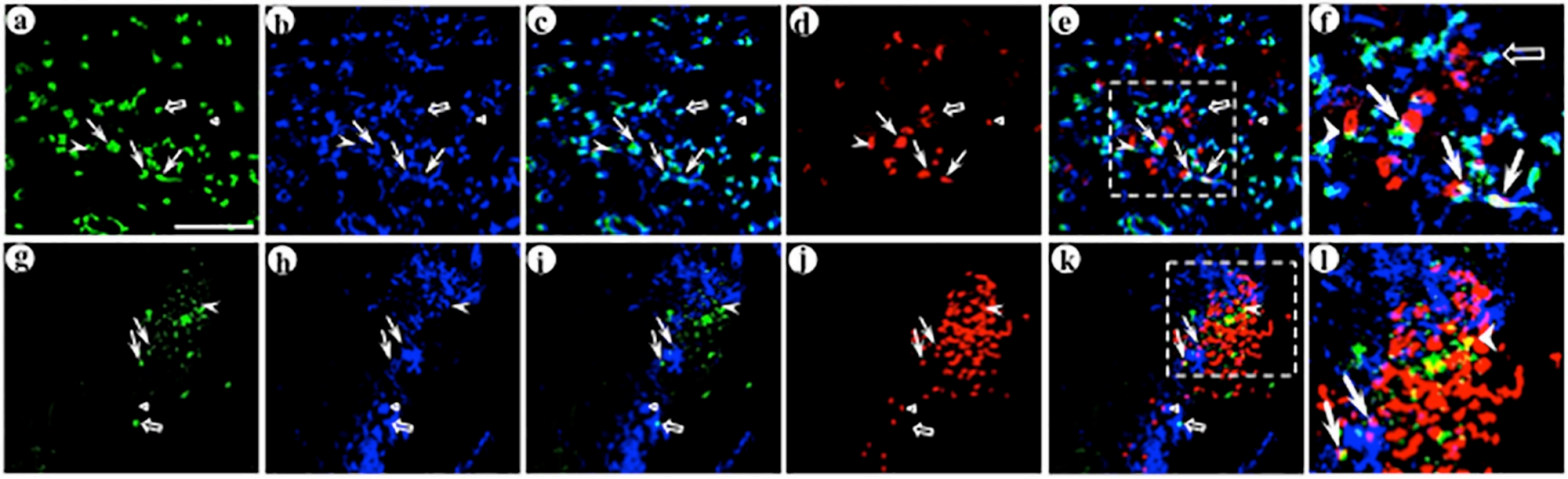
The synthetic peptide neurotransmitter dcv-secretogranin/anti-GABA_A_ß2or3-HtoL/his-tag supports gene transfer to the calretinin-containing subtype of GABAergic neurons. The experimental design and immunofluorescent costaining were as above, but calretinin-IR was assayed instead of parvalbumin-IR. (A-F) Image stacks show neuronal morphology for the transduced postsynaptic dendrites and cell bodies, proximal to transduced axons; (A) His tag-IR (fluorescein-conjugated secondary antibody), (B) GFP-IR (Alexa Fluor 633-conjugated secondary antibody), (C) merge of His tag-IR and GFP-IR, (D) calretinin-IR (TRITC-conjugated secondary antibody), (E) merge of all three IR, (F) merge, an enlarged area showing adjacent transduced axons and dendrites that contain calretinin-IR. Arrows, transduced axons proximal to transduced dendrites, with calretinin-IR. Open arrow, a transduced axon proximal to a transduced dendrite that lacks calretinin-IR. Arrowheads, transduced axons distant from transduced dendrites, but proximal to calretinin-IR. Open triangle, a transduced dendrite that contains calretinin-IR, but distant from a transduced axon. (G-L) Single confocal images in each channel show that delivery of this presynaptic vector, followed by the targeted postsynaptic vector, supported gene transfer across GABA_A_ ß2- or ß3-containing synapses to calretinin-containing postsynaptic neurons; (G) His tag-IR, (H) GFP-IR, (I) merge of His tag-IR and GFP-IR, (J) calretinin-IR, (K) merge of all three IR, (L) merge, an enlarged area showing adjacent transduced axons and dendrites that contain calretinin-IR. Panels G-L show a different field than for panels A-F. Scale bar: 20 μm.

**Fig 10:**
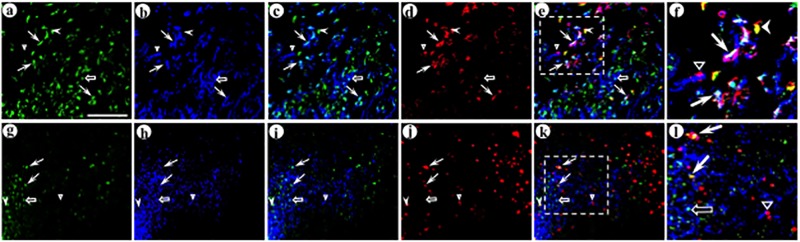
This synthetic peptide neurotransmitter supports gene transfer to the calbindin-containing subtype of GABAergic neurons. The experimental design and immunofluorescent costaining were as above, but calbindin-IR was assayed instead of parvalbumin-IR. (A-F) Image stacks show neuronal morphology for the transduced postsynaptic dendrites and cell bodies, proximal to transduced axons; (A) His tag-IR (fluorescein-conjugated secondary antibody), (B) GFP-IR (Alexa Fluor 633-conjugated secondary antibody), (C) merge of His tag-IR and GFP-IR, (D) calbindin-IR (TRITC-conjugated secondary antibody), (E) merge of all three IR, (F) merge, an enlarged area showing adjacent transduced axons and dendrites that contain calbindin-IR. Arrow, a transduced axon proximal to a transduced dendrite, with calbindin-IR. Open arrow, a transduced axon proximal to a transduced dendrite that lacks calbindin-IR. Arrowhead, a transduced axon distant from a transduced dendrite, but proximal to calbindin-IR. Open triangle, a transduced dendrite that contains calbindin-IR, but distant from a transduced axon. (G-L) Single confocal images in each channel show that delivery of the presynaptic vector, followed by the targeted postsynaptic vector, supported gene transfer across GABA_A_ ß2- or ß3-containing synapses to calbindin-containing postsynaptic neurons; (G) His tag-IR, (H) GFP-IR, (I) merge of His tag-IR and GFP-IR, (J) calbindin-IR, (K) merge of all three IR, (L) merge, an enlarged area showing adjacent transduced axons and dendrites that contain calbindin-IR. Panels G-L show a different field than for panels A-F. Scale bar: 20 μm.

As dcv-secretogranin/anti-GABA_A_ß2or3-HtoL/his-tag was designed to support gene transfer selectively across GABA_A_ ß2- or ß3-containing synapses, and the ß2 and ß3 subunits are widely expressed in neocortical neurons, we examined the subtypes of GABAergic neurons that received the postsynaptic vector. GABAergic neurons are frequently partitioned into three subtypes, based on the calcium binding protein they express, parvalbumin, or calretinin, or calbindin. Gene transfer was performed as above, and gene transfer to connected neurons was assayed as above, but the immunofluorescent assay contained a third primary antibody to characterize the GABAergic subtype (these triple staining assays used different secondary antibodies/colors than the double staining assays described above). Photomicrographs were first analyzed to identify the synapses that supported gene transfer to connected neurons (as above), and then these synapses were examined for containing, or lacking, a specific calcium binding protein. For each GABAergic subtype, to visualize neuronal morphology, we analyzed image stacks of photomicrographs; and to quantify synapses between transduced axons and dendrites or cell bodies, we analyzed single images in each fluorescent channel, as above. Photomicrographs showed that many of the postsynaptic neurons contained parvalbumin (Fig 8), and some of the postsynaptic neurons contained calretinin (Fig 9) or calbindin (Fig 10). Counts of this data were performed; an example of these counts is shown in S5B Fig, and S5B Fig legend details the procedure. The results showed that for gene transfer across GABA_A_ ß2- or ß3-containing synapses, 51% of the postsynaptic neurons contained parvalbumin, 31% of these neurons contained calretinin, and 24% contained calbindin (Table 3). These markers defined three subtypes of GABAergic neurons for the postsynaptic gene transfer (F_2,6_ = 1,009 p<0.001. Two way comparisons: parvalbumin vs. calretinin or calbindin, or calretinin vs. calbindin, all p<0.001). Together, these three GABAergic neuron markers appear to account for essentially all of the gene transfer to postsynaptic neurons, 106%. We did not assay postsynaptic glutamatergic neurons, as the two commonly used markers for glutamatergic neurons are presynaptic markers (phosphate-activated glutaminase or a vesicular glutamate transporter (VGLUT1 or VGLUT2)). Of note, the antibody that forms the foundation for the synthetic peptide neurotransmitters recognizes both GABA_A_ ß2 and ß3 subunits [22–24]. Interestingly, the ß2 subunit is preferentially expressed on GABAergic neurons, and the ß3 subunit is preferentially expressed on glutamatergic neurons ([53] http://linnarssonlab.org/cortex/). It is not known if this antibody, or this synthetic peptide neurotransmitter, display preferential binding to either the ß2 or ß3 subunit.

**Table 3:** GABAergic subtype markers in the connected postsynaptic neurons after gene transfer across GABA_A_ ß2 or ß3-containing synapses.

Presynaptic vector	Synapse marker	His tag-IR and GFP-IR synapses	% Triple staining
pGADdcv-secretogranin/anti-GABAAß2or3-HtoL/his-tag	Parvalbumin	466±162	51±0
	Calretinin	148±16	31±0
	Calbindin	231±11	24±0

In each section that was examined, all the transduced presynaptic axons that were adjacent to a transduced postsynaptic dendrite were identified. These synapses were scored for containing or lacking the indicated marker. Three rats per condition; three or four sections were scored for each condition; mean±s.e.m. are shown.

## Discussion

We have developed a technology to selectively deliver different genes into pre- or post-synaptic local interneurons that are connected by GABAergic synapses. This synapse type selectivity is conferred by a synthetic peptide neurotransmitter that is expressed in the presynaptic neurons, and has three domains: Processing and release of the recombinant protein as a peptide neurotransmitter is supported by the N-terminal, DCV sorting domain; the middle domain binds to GABA_A_ receptors on the postsynaptic neurons; and a His tag is the C-terminal domain. Upon release, this synthetic peptide neurotransmitter binds to postsynaptic neurons that contain GABA_A_ receptors. The second gene transfer is selective for these neurons, and uses antibody-mediated, targeted gene transfer and anti-His tag antibodies.

We used ScFv to bind to postsynaptic GABA_A_ receptors, and use of ScFv defines a novel class of synthetic peptide neurotransmitters that have some attractive properties. These synthetic peptide neurotransmitters may support gene transfer across a wide range of synapse types, as ScFv can be developed from most antibodies [25]. The two synthetic peptide neurotransmitters studied here contained different ScFv; one ScFv placed the V_H_ region N-terminal to the V_L_ region, and the other ScFv inverted the order of these regions. Only one of these synthetic peptide neurotransmitters supported gene transfer selectively across GABAergic synapses, and the basis for this difference is not known. These synthetic peptide neurotransmitters contain a spacer between each of the domains; thus, it appears unlikely that defective assembly of the ScFv or steric hindrance between domains can account for the different capabilities of these two synthetic peptide neurotransmitters. The technology developed here may be supported by a number of virus vector systems, as most virus vectors can express a synthetic peptide neurotransmitter, and antibody-mediated, targeted gene transfer has been established for many virus vectors, including Sindbis Virus, Adenovirus, classical retrovirus, Lentivirus, Adeno-Associated Virus (AAV), and HSV-1 vectors [40, 54–61].

This technology supports efficient gene transfer across multiple types of GABAergic synapses. Using the preferred synthetic peptide neurotransmitter and targeted gene transfer, 61% of the transduced presynaptic axons were connected to a transduced postsynaptic dendrite. In contrast, for the comparison, untargeted gene transfer, only 21% of the transduced presynaptic axons were connected to a transduced postsynaptic dendrite. Further, this synthetic peptide neurotransmitter supports gene transfer to the three major subtypes of GABAergic neurons, as defined by the calcium binding proteins parvalbumin, calretinin, and calbindin. Additional specificity for the pre- or post-synaptic neuron type, or subtypes of GABAergic synapses, is available. Here, the presynaptic gene transfer used standard, untargeted gene transfer, but this gene transfer could use antibody mediated targeting to confer selectivity for a specific neuron type [40, 41, 62]. Further, the postsynaptic vector contained a pan-neuronal promoter for quantifying gene transfer to connected neurons, but postsynaptic neuron type specificity could be conferred by using a neuron type-specific promoter, such as a glutamatergic- or GABAergic-specific promoter [26]. Thus, improvements in the specificity of gene transfer could be realized by using the preferred synthetic peptide neurotransmitter developed here in combination with targeted gene transfer for the presynaptic gene transfer and/or a neuron type-specific promoter in the postsynaptic vector. Additionally, here GABAergic synapse specificity was achieved by targeting GABA_A_ ß2 and ß3, present in many GABAergic synapses, but the postsynaptic gene transfer could be restricted to specific subtypes of GABAergic synapses by targeting rare subunits of GABA_A_ receptors or specific neurotransmitter receptors that define GABAergic subtypes.

Here, the presynaptic vector was injected into the middle neocortical layers, the postsynaptic vector was injected into the upper neocortical layers, and gene transfer was predominately to connected, postsynaptic GABAergic neurons. The molecular basis for the observed specificity for postsynaptic GABAergic, rather than glutamatergic, neurons is not clear. The synthetic peptide neurotransmitter is based on an antibody that recognizes both GABA_A_ ß2 and ß3 subunits [22–24]; ß2 is preferentially expressed in GABAergic neurons; and ß3 is preferentially expressed in glutamatergic neurons ([53] http://linnarssonlab.org/cortex/). Thus, the synthetic peptide neurotransmitter might preferentially bind to ß2 subunits on GABAergic neurons. Another possibility is that many glutamatergic cell bodies are located in the lower neocortical layers; and the synthetic peptide neurotransmitter might preferentially bind to the cell bodies of GABAergic neurons in the upper neocortical layers rather than to the dendrites of glutamatergic neurons. Many GABAergic neurons form synapses onto glutamatergic neurons, including many in the middle and lower neocortical layers [63]. Thus, injecting the pre- and post-synaptic vectors into the middle or lower neocortical layers might support gene transfer to other connected neurons that are part of local neocortical circuits.

Most genetic approaches for mapping or modifying forebrain circuits transduce neurons in different forebrain areas that are connected by excitatory, glutamatergic synapses; in contrast, here we developed technology to selectively transduce local interneurons, within a neocortical area, that are connected by inhibitory, GABAergic synapses. As the cell bodies of connected interneurons are in close proximity, transducing connected local interneurons poses special challenges for both the gene transfer and the assays. Further, as GABAergic and glutamatergic synapses and postsynaptic specializations contain different proteins [6], strategies that are effective for glutamatergic synapses may not be applicable to GABAergic synapses. Moreover, as GABAergic synapses represent a relatively small fraction, 5–15%, of neocortical synapses [6], targeting gene transfer across these synapses may be more difficult. Here, a synthetic peptide neurotransmitter containing a ScFv anti-GABA_A_ ß2 or ß3 supported 61% targeting across GABAergic synapses; in contrast, synthetic peptide neurotransmitters containing ScFv anti-NR2B or ScFv anti-mGluR5 supported up to 82% targeting across those specific glutamatergic synapse types [64, 65]. Also, we added Brain-Derived Neurotrophic Factor (BDNF) to a synthetic peptide neurotransmitter, and obtained 73% targeting across synapses that contain *TrkB* [66]. Using triple staining for a synthetic peptide neurotransmitter (His tag), expression in the postsynaptic neuron (GFP), and the cognate receptor (NR2B, mGluR5, or *TrkB*), we showed that each of these three synthetic peptide neurotransmitters is 95 to 97% specific for synapses that contain the cognate receptor. Here, we did not quantify the percentage of targeted synapses that contain GABA_A_ receptors, but we did establish that GABAergic neuron types account for the clear majority of the postsynaptic neurons. Together, these results show that specific synthetic peptide neurotransmitters can be highly specific for the cognate receptor and synapse type, and the level of specificity depends upon the specific synthetic peptide neurotransmitter. Improved targeting across GABAergic synapses might be achieved by targeting different subunits of GABA_A_ receptors, or other receptors present in specific GABAergic synapse types. Nonetheless, 61% targeting is a high level, and may support physiological studies.

The capability to deliver different genes into pre- and post-synaptic neurons connected by a GABAergic synapse has multiple applications to basic neuroscience and neurology. Local circuits might be mapped in detail by expressing different genes in connected neurons, such as specific fluorescent proteins. This new gene transfer technology may enable novel studies on information processing in local neocortical circuits, including studies on synaptic plasticity, circuit physiology, and learning. For example, we established a genetic intervention that targets some of the essential information for specific visual discriminations to the transduced neurons in POR cortex [10, 17]. The gene transfer for these studies used the same stereotactic coordinates as for the presynaptic gene transfer in the present study. Thus, the connected GABAergic interneurons in the upper layers of POR cortex are likely to have important roles in this learning. The role of these neurons in this learning might be studied by first activating PKC pathways in the presynaptic neurons in POR cortex, and then using the present technology to alter neuronal activity, or specific signaling or transcriptional pathways, in these postsynaptic interneurons. Established technology that might support such studies include using a specific siRNA to inhibit expression of a gene, CRISPR/cas9 derivatives to activate or inhibit gene expression, or optogenetics or other genetic tools to activate or block neuronal activity. Different components of the local circuit in POR cortex might be studied by using different injection sites for the postsynaptic gene transfer, and by inserting different promoters into the postsynaptic vectors. Moreover, this new gene transfer technology may have applications to understanding and treating specific neurological disorders. Particular neocortical GABAergic neurons and synapses play critical roles in specific disorders, including schizophrenia and autism [1]. Thus, gene therapy treatments for these conditions might be based on the capability to alter neurotransmission at specific GABAergic synapses.

## Supporting information

S1 Fig Experimentally determined anti-GABA_A_ ß2/3 light chain sequence. (PDF)

S2 Fig Experimentally determined anti-GABA_A_ ß2/3 heavy chain sequence. (PDF)

S3 Fig The DNA sequence for the synthetic peptide neurotransmitter dcv-secretogranin/anti-GABA_A_ß2/3-HtoL/his-tag. (PDF)

S4 Fig The DNA sequence for the synthetic peptide neurotransmitter dcv-pomc/anti-GABA_A_ß2/3-LtoH/his-tag. (PDF)

S5 Fig Quantification of (A) the efficiency of gene transfer to connected neurons, or (B) the percentage of transduced postsynaptic neurons that also contain parvalbumin. (A) Following gene transfer to connected neurons, labeling and counting of transduced axons that are proximal to, or distant from, a transduced dendrite. The experimental design and vectors followed Fig 3. The upper layers of POR cortex were examined. The photomicrograph shows a merge of the transduced axons (His tag-IR; Texas red-conjugated secondary antibody) and the transduced dendrites (GFP-IR; FITC-conjugated secondary antibody). Each transduced axon that is proximal to a transduced dendrite was labeled with a “+”; this image contains 79 of these axons. Inversely, each transduced axon that is distant from a transduced dendrite was labeled with a “-”; this image contains 12 of these axons. The targeting efficiency for the modest sample in this image is 79 / (79+12), or 87%. Multiple images, from multiple rats, were analyzed in this manner to generate the data in Table 2. (B) Following gene transfer to connected neurons, labeling and counting of the connected postsynaptic neurons that contain, or lack, parvalbumin. The experimental design and vectors followed Fig 8. The upper layers of POR cortex were examined. The photomicrograph shows a merge of the transduced axons (His tag-IR; fluorescein-conjugated secondary antibody), the transduced dendrites (GFP-IR; Alexa Fluor 633-conjugated secondary antibody), and parvalbumin-IR (TRITC-conjugated secondary antibody). The synapses that supported gene transfer to connected neurons were identified as in panel A and labeled with a “+”; this image contains 49 connected, transduced axons and dendrites. The postsynaptic neurons that also contain parvalbumin were scored by adding a “$”; this image contains 17 postsynaptic neurons that also contain parvalbumin. The percentage of postsynaptic neurons that also contain parvalbumin for the modest sample in this image is 17 / 49, or 35%. Multiple images, from multiple rats, were analyzed in this manner to generate the data in Table 3. Scale bar: 50 μm.
(PDF)

## References

[1] Marin O. Interneuron dysfunction in psychiatric disorders. Nat Rev Neurosci. 2012;13(2):107–20. Epub 2012/01/19. 10.1038/nrn3155 . 22251963

[2] Hubel DH, Wiesel TN. Ferrier lecture. Functional architecture of macaque monkey visual cortex. Proc Royal Soc London—Series B. 1977;198:1–59. 10.1098/rspb.1977.0085 20635

[3] Hubel DH, Wiesel TN. Receptive fields, binocular interaction and functional architecture in the cat's visual cortex. J Physiol. 1962;160:106–54. 10.1113/jphysiol.1962.sp006837 14449617 PMC1359523

[4] Dudai Y. The Neurobiology of Memory. Oxford, England: Oxford Univ. Press; 1989.

[5] Kubota Y, Karube F, Nomura M, Kawaguchi Y. The Diversity of Cortical Inhibitory Synapses. Frontiers in neural circuits. 2016;10:27 Epub 2016/05/21. 10.3389/fncir.2016.00027 . 27199670 PMC4842771

[6] Heller EA, Zhang W, Selimi F, Earnheart JC, Slimak MA, Santos-Torres J, et al The biochemical anatomy of cortical inhibitory synapses. PLoS One. 2012;7(6):e39572 Epub 2012/07/07. 10.1371/journal.pone.0039572 . 22768092 PMC3387162

[7] Le Magueresse C, Monyer H. GABAergic interneurons shape the functional maturation of the cortex. Neuron. 2013;77(3):388–405. Epub 2013/02/12. . 23395369 10.1016/j.neuron.2013.01.011

[8] Fenno L, Yizhar O, Deisseroth K. The development and application of optogenetics. Annu Rev Neurosci. 2011;34:389–412. Epub 2011/06/23. 10.1146/annurev-neuro-061010-113817 . 21692661 PMC6699620

[9] Luo L, Callaway EM, Svoboda K. Genetic dissection of neural circuits. Neuron. 2008;57(5):634–60. Epub 2008/03/18. 10.1016/j.neuron.2008.01.002 . 18341986 PMC2628815

[10] Zhang G, Wang X, Kong L, Lu X, Lee B, Liu M, et al Genetic enhancement of visual learning by activation of protein kinase C pathways in small groups of rat cortical neurons. J Neurosci. 2005;25:8468–81. 10.1523/JNEUROSCI.2271-05.2005 . 16162929 PMC2581869

[11] Dymecki SM, Kim JC. Molecular neuroanatomy's "Three Gs": a primer. Neuron. 2007;54(1):17–34. Epub 2007/04/06. . 17408575 10.1016/j.neuron.2007.03.009 PMC2897592

[12] Kim J, Zhao T, Petralia RS, Yu Y, Peng H, Myers E, et al mGRASP enables mapping mammalian synaptic connectivity with light microscopy. Nat Methods. 2011;9:96–102. Epub 2011/12/06. 10.1038/nmeth.1784 . 22138823 PMC3424517

[13] Feng L, Kwon O, Lee B, Oh WC, Kim J. Using mammalian GFP reconstitution across synaptic partners (mGRASP) to map synaptic connectivity in the mouse brain. Nat Protoc. 2014;9(10):2425–37. Epub 2014/09/19. 10.1038/nprot.2014.166 . 25232938

[14] Lo L, Anderson DJ. A cre-dependent, anterograde transsynaptic viral tracer for mapping output pathways of genetically marked neurons. Neuron. 2011;72(6):938–50. Epub 2011/12/27. 10.1016/j.neuron.2011.12.002 . 22196330 PMC3275419

[15] Osakada F, Mori T, Cetin AH, Marshel JH, Virgen B, Callaway EM. New rabies virus variants for monitoring and manipulating activity and gene expression in defined neural circuits. Neuron. 2011;71(4):617–31. Epub 2011/08/27. 10.1016/j.neuron.2011.07.005 . 21867879 PMC3189471

[16] Murray EA, Bussey TJ, Saksida LM. Visual Perception and Memory: A New View of Medial Temporal Lobe Function in Primates and Rodents. Annu Rev Neurosci. 2007;30:99–122. 10.1146/annurev.neuro.29.051605.113046 . 17417938

[17] Zhang G, Cao H, Kong L, O’Brien J, Baughns A, Jan M, et al Identified circuit in rat postrhinal cortex encodes essential information for performing specific visual shape discriminations. Proc Natl Acad Sci USA. 2010;107:14478–83. 10.1073/pnas.0912950107 . 20660720 PMC2922527

[18] Burwell RD, Amaral DG. Cortical afferents of the perirhinal, postrhinal, and entorhinal cortices of the rat. J Comp Neurol. 1998;398(2):179–205. 9700566 10.1002/(sici)1096-9861(19980824)398:2<179::aid-cne3>3.0.co;2-y

[19] Agster KL, Burwell RD. Cortical efferents of the perirhinal, postrhinal, and entorhinal cortices of the rat. Hippocampus. 2009;19(12):1159–86. Epub 2009/04/11. 10.1002/hipo.20578 . 19360714 PMC3066185

[20] Zhang G, Zhao H, Choi EM, Svestka M, Wang X, Cook RG, et al CaMKII, MAPK, and CREB are coactivated in identified neurons in a cortical circuit required for performing visual shape discriminations. Hippocampus. 2012;22(12):2276–89. 10.1002/hipo.22045 . 22736516

[21] Zhang G, Zhao H, Cao H, Li X, Geller AI. Targeted gene transfer of different genes to presynaptic and postsynaptic neocortical neurons connected by a glutamatergic synapse. Brain Res. 2012;1473:173–84. 10.1016/j.brainres.2012.07.024 . 22820303 PMC3442772

[22] Vitorica J, Park D, Chin G, de Blas AL. Monoclonal antibodies and conventional antisera to the GABAA receptor/benzodiazepine receptor/Cl- channel complex. J Neurosci. 1988;8(2):615–22. Epub 1988/02/01. . 2828566 10.1523/JNEUROSCI.08-02-00615.1988 PMC6569298

[23] de Blas AL, Vitorica J, Friedrich P. Localization of the GABAA receptor in the rat brain with a monoclonal antibody to the 57,000 Mr peptide of the GABAA receptor/benzodiazepine receptor/Cl- channel complex. J Neurosci. 1988;8(2):602–14. Epub 1988/02/01. . 2828565 10.1523/JNEUROSCI.08-02-00602.1988 PMC6569288

[24] Ewert M, de Blas AL, Mohler H, Seeburg PH. A prominent epitope on GABAA receptors is recognized by two different monoclonal antibodies. Brain Res. 1992;569(1):57–62. Epub 1992/01/08. . 1377081 10.1016/0006-8993(92)90368-j

[25] Huston JS, Mudgett-Hunter M, Tai MS, McCartney J, Warren F, Haber E, et al Protein engineering of single-chain Fv analogs and fusion proteins. Methods Enzymol. 1991;203:46–88. Epub 1991/01/01. . 1762568 10.1016/0076-6879(91)03005-2

[26] Rasmussen M, Kong L, Zhang G, Liu M, Wang X, Szabo G, et al Glutamatergic or GABAergic neuron-specific, long-term expression in neocortical neurons from helper virus-free HSV-1 vectors containing the phosphate-activated glutaminase, vesicular glutamate transporter-1, or glutamic acid decarboxylase promoter. Brain Res. 2007;1144:19–32. 10.1016/j.brainres.2007.01.125 . 17331479 PMC2694742

[27] Kobayashi T, Ebihara S, Ishii K, Nishijima M, Endo S, Takaku A, et al Structural and functional characterization of mouse glutamate decarboxylase 67 gene promoter. Biochim Biophys Acta. 2003;1628(3):156–68. . 12932828 10.1016/s0167-4781(03)00138-6

[28] Kozak M. Point mutations define a sequence flanking the AUG initiator codon that modulates translation by eukaryotic ribosomes. Cell. 1986;44(2):283–92. Epub 1986/01/31. . 3943125 10.1016/0092-8674(86)90762-2

[29] Nakagawa S, Niimura Y, Gojobori T, Tanaka H, Miura K. Diversity of preferred nucleotide sequences around the translation initiation codon in eukaryote genomes. Nucleic Acids Res. 2008;36(3):861–71. Epub 2007/12/19. 10.1093/nar/gkm1102 . 18086709 PMC2241899

[30] Courel M, Vasquez MS, Hook VY, Mahata SK, Taupenot L. Sorting of the neuroendocrine secretory protein Secretogranin II into the regulated secretory pathway: role of N- and C-terminal alpha-helical domains. J Biol Chem. 2008;283(17):11807–22. Epub 2008/02/27. 10.1074/jbc.M709832200 . 18299326 PMC2431052

[31] Gerdes HH, Rosa P, Phillips E, Baeuerle PA, Frank R, Argos P, et al The primary structure of human secretogranin II, a widespread tyrosine-sulfated secretory granule protein that exhibits low pH- and calcium-induced aggregation. J Biol Chem. 1989;264(20):12009–15. Epub 1989/07/15. . 2745426

[32] Cool DR, Loh YP. Identification of a sorting signal for the regulated secretory pathway at the N-terminus of pro-opiomelanocortin. Biochimie. 1994;76(3–4):265–70. Epub 1994/01/01. . 7819333 10.1016/0300-9084(94)90156-2 PMC7131109

[33] Cool DR, Fenger M, Snell CR, Loh YP. Identification of the sorting signal motif within pro-opiomelanocortin for the regulated secretory pathway. J Biol Chem. 1995;270(15):8723–9. Epub 1995/04/14. 10.1074/jbc.270.15.8723 . 7721777

[34] Zhang G, Zhao H, Cao H, Geller AI. Overexpression of either lysine-specific demethylase-1 or CLOCK, but not Co-Rest, improves long-term expression from a modified neurofilament promoter, in a helper virus-free HSV-1 vector system. Brain Res. 2012;1436:157–67. 10.1016/j.brainres.2011.12.011 . 22208646 PMC3287058

[35] Kameda H, Furuta T, Matsuda W, Ohira K, Nakamura K, Hioki H, et al Targeting green fluorescent protein to dendritic membrane in central neurons. Neurosci Res. 2008;61(1):79–91. Epub 2008/03/18. 10.1016/j.neures.2008.01.014 . 18342383

[36] Song S, Wang Y, Bak SY, During MJ, Bryan J, Ashe O, et al Modulation of rat rotational behavior by direct gene transfer of constitutively active protein kinase C into nigrostriatal neurons. J Neurosci. 1998;18(11):4119–32. 9592092 10.1523/JNEUROSCI.18-11-04119.1998 PMC6792804

[37] Smith IL, Hardwicke MA, Sandri-Goldin RM. Evidence that the herpes simplex virus immediate early protein ICP27 acts post-transcriptionally during infection to regulate gene expression. Virology. 1992;186(1):74–86. 1309283 10.1016/0042-6822(92)90062-t

[38] Fraefel C, Song S, Lim F, Lang P, Yu L, Wang Y, et al Helper virus-free transfer of herpes simplex virus type 1 plasmid vectors into neural cells. J Virol. 1996;70(10):7190–7. 8794366 10.1128/jvi.70.10.7190-7197.1996 PMC190772

[39] Sun M, Zhang GR, Yang T, Yu L, Geller AI. Improved titers for helper virus-free herpes simplex virus type 1 plasmid vectors by optimization of the packaging protocol and addition of noninfectious herpes simplex virus-related particles (previral DNA replication enveloped particles) to the packaging procedure. Hum Gene Ther. 1999;10(12):2005–11. 10.1089/10430349950017365 10466634

[40] Cao H, Zhang GR, Geller AI. Antibody-mediated targeted gene transfer to NMDA NR1-containing neurons in rat neocortex by helper virus-free HSV-1 vector particles containing a chimeric HSV-1 glycoprotein C—Staphylococcus A protein. Brain Res. 2010;1351:1–12. 10.1016/j.brainres.2010.06.045 . 20599821 PMC2929402

[41] Cao H, Zhang GR, Geller AI. Antibody-mediated targeted gene transfer of helper virus-free HSV-1 vectors to rat neocortical neurons that contain either NMDA receptor 2A or 2B subunits. *Brain Research*. 2011;1415:127–35. 10.1016/j.brainres.2011.08.010 . 21885042 PMC3176983

[42] Gao Q, Sun M, Wang X, Geller AI. Isolation of an enhancer from the rat tyrosine hydroxylase promoter that supports long-term, neuronal-specific expression from a neurofilament promoter, in a helper virus-free HSV-1 vector system. Brain Res. 2007;1130:1–16. 10.1016/j.brainres.2006.10.018 . 17169349 PMC2694737

[43] Yang T, Zhang G, Zhang W, Sun M, Wang X, Geller AI. Enhanced reporter gene expression in the rat brain from helper virus-free HSV-1 vectors packaged in the presence of specific mutated HSV-1 proteins that affect the virion. Molec Brain Res. 2001;90:1–16. 11376851 10.1016/s0169-328x(01)00059-6

[44] Zhang G, Wang X, Yang T, Sun M, Zhang W, Wang Y, et al A tyrosine hydroxylase—neurofilament chimeric promoter enhances long-term expression in rat forebrain neurons from helper virus-free HSV-1 vectors. Molec Brain Res. 2000;84:17–31. 11113528 10.1016/s0169-328x(00)00197-2

[45] Paxinos G, Watson C. The rat brain in stereotaxic coordinates. Sidney: Academic Press; 1986. 10.1016/0165-0270(80)90021-7 6110810

[46] Dikeakos JD, Reudelhuber TL. Sending proteins to dense core secretory granules: still a lot to sort out. J Cell Biol. 2007;177(2):191–6. Epub 2007/04/18. 10.1083/jcb.200701024 . 17438078 PMC2064127

[47] Moskal JR, Kuo AG, Weiss C, Wood PL, O'Connor Hanson A, Kelso S, et al GLYX-13: a monoclonal antibody-derived peptide that acts as an N-methyl-D-aspartate receptor modulator. Neuropharmacology. 2005;49(7):1077–87. 10.1016/j.neuropharm.2005.06.006 . 16051282

[48] Moskal JR, Yamamoto H, Colley PA. The use of antibody engineering to create novel drugs that target N-methyl-D-aspartate receptors. Curr Drug Targets. 2001;2(3):331–45. . 11554557 10.2174/1389450013348399

[49] Burwell RD, Amaral DG. Perirhinal and postrhinal cortices of the rat: interconnectivity and connections with the entorhinal cortex. J Comp Neurol. 1998;391(3):293–321. 9492202 10.1002/(sici)1096-9861(19980216)391:3<293::aid-cne2>3.0.co;2-x

[50] Spear PG, Longnecker R. Herpesvirus entry: an update. J Virol. 2003;77(19):10179–85. 10.1128/JVI.77.19.10179-10185.2003 . 12970403 PMC228481

[51] Sun M, Kong L, Wang X, Holmes C, Gao Q, Zhang W, et al Coexpression of tyrosine hydroxylase, GTP cyclohydrolase I, aromatic amino acid decarboxylase, and vesicular monoamine transporter 2 from a helper virus-free HSV-1 vector supports high-evel, long-term biochemical and behavioral correction of a rat model of Parkinson’s disease. Hum Gene Ther. 2004;15:1177–96. 10.1089/hum.2004.15.1177 . 15684695 PMC2581868

[52] Zhang G, Cao H, Li X, Zhao H, Geller AI. Genetic labeling of both the axons of transduced, glutamatergic neurons in rat postrhinal cortex and their postsynaptic neurons in other neocortical areas by Herpes Simplex Virus vectors that coexpress an axon-targeted ß-galactosidase and wheat germ agglutinin from a vesicular glutamate transporter-1 promoter. Brain Res. 2010;1361:1–11. 10.1016/j.brainres.2010.09.030 . 20849834 PMC2963663

[53] Zeisel A, Munoz-Manchado AB, Codeluppi S, Lonnerberg P, La Manno G, Jureus A, et al Brain structure. Cell types in the mouse cortex and hippocampus revealed by single-cell RNA-seq. Science. 2015;347(6226):1138–42. Epub 2015/02/24. 10.1126/science.aaa1934 . 25700174

[54] Morizono K, Bristol G, Xie YM, Kung SK, Chen IS. Antibody-directed targeting of retroviral vectors via cell surface antigens. J Virol. 2001;75(17):8016–20. 10.1128/JVI.75.17.8016-8020.2001 . 11483746 PMC115045

[55] Morizono K, Chen IS. Targeted gene delivery by intravenous injection of retroviral vectors. Cell Cycle. 2005;4(7):854–6. 10.4161/cc.4.7.1789 . 15970695

[56] Morizono K, Xie Y, Ringpis GE, Johnson M, Nassanian H, Lee B, et al Lentiviral vector retargeting to P-glycoprotein on metastatic melanoma through intravenous injection. Nat Med. 2005;11(3):346–52. 10.1038/nm1192 . 15711560

[57] Bergman I, Whitaker-Dowling P, Gao Y, Griffin JA, Watkins SC. Vesicular stomatitis virus expressing a chimeric Sindbis glycoprotein containing an Fc antibody binding domain targets to Her2/neu overexpressing breast cancer cells. Virology. 2003;316(2):337–47. . 14644615 10.1016/j.virol.2003.07.010

[58] Ried MU, Girod A, Leike K, Buning H, Hallek M. Adeno-associated virus capsids displaying immunoglobulin-binding domains permit antibody-mediated vector retargeting to specific cell surface receptors. J Virol. 2002;76(9):4559–66. 10.1128/JVI.76.9.4559-4566.2002 . 11932421 PMC155067

[59] Ohno K, Sawai K, Iijima Y, Levin B, Meruelo D. Cell-specific targeting of Sindbis virus vectors displaying IgG-binding domains of protein A. Nat Biotechnol. 1997;15(8):763–7. 10.1038/nbt0897-763 . 9255791

[60] Volpers C, Thirion C, Biermann V, Hussmann S, Kewes H, Dunant P, et al Antibody-mediated targeting of an adenovirus vector modified to contain a synthetic immunoglobulin g-binding domain in the capsid. J Virol. 2003;77(3):2093–104. 10.1128/JVI.77.3.2093-2104.2003 . 12525644 PMC140881

[61] Tai CK, Logg CR, Park JM, Anderson WF, Press MF, Kasahara N. Antibody-mediated targeting of replication-competent retroviral vectors. Hum Gene Ther. 2003;14(8):789–802. 10.1089/104303403765255174 . 12804141

[62] Wang X, Kong L, Zhang G, Sun M, Geller AI. Targeted gene transfer to nigrostriatal neurons in the rat brain by helper virus-free HSV-1 vector particles that contain either a chimeric HSV-1 glycoprotein C—GDNF or a gC—BDNF protein. Molec Brain Res. 2005;139:88–102. 10.1016/j.molbrainres.2005.05.029 . 15993510 PMC2581866

[63] Jiang X, Shen S, Cadwell CR, Berens P, Sinz F, Ecker AS, et al Principles of connectivity among morphologically defined cell types in adult neocortex. Science. 2015;350(6264):aac9462. Epub 2015/11/28. 10.1126/science.aac9462 . 26612957 PMC4809866

[64] Nagayach A, Singh A, Geller AI. Delivery of different genes into pre- and post-synaptic neocortical neurons connected by mGluR5-containing synapses. Journal of Molecular Neuroscience. 2019;IN PRESS. 10.1007/s12031-019-01317-9 PMC6615967 30972540

[65] Nagayach A, Ghafari M, Singh A, Geller AI. Delivery of different genes into pre- and post-synaptic neocortical neurons connected by specific synapse types that support learning. Submitted. 2019.

[66] Nagayach A, Singh A, Geller AI. Delivery of different genes into presynaptic and postsynaptic neocortical neurons connected by a BDNF-TrkB synapse. Brain Research. 2019;1712:16–24. 10.1016/j.brainres.2019.01.038 30710509

