# Supplementary material for: Delivery of different genes into pre- and post-synaptic neocortical interneurons connected by GABAergic synapses
Source: PLoS One. 2019 May 24;14(5):e0217094. doi: 10.1371/journal.pone.0217094 (PMC6534327; doi:10.1371/journal.pone.0217094)
Supplement: S2 Fig — (PDF) [file pone.0217094.s002.pdf]

GACCAGGCATCCCAGGGTCACCATGGAGTTAGTTTGGGCAGCAGATCCAGGGGCCAGTGGATA

GACAGATGGGGGTGTCGTTTTGGCTGAGGAGACTGTAAGAGTGGTGCCTTGGCCCCAGTGTTCA

TCCCAGGCCCATTTTGCACAGAAATAGACCGCAGAGTCCACAGAGGTCAGGCTGCTGAGCTCCA

TGTAGGCTGTGCTGGAGGATTTGTCTGCAGTCAGTGTGGCCTTGTCCTTGAACTTCCCATTGTAC

TTAACATCTCCATTTCCAGGATGAATCCGTCCAATCCACTCAAGACCCTGTCCAGGCCTCTGTTTC

ACCCAGTTCATCCAAGAACTACTGAATTCGTAGCCAGAAACTTTGCAGGAAATCTTCACTGAGGC

CCCAGGCTTCACCAGCTCAGGTCCTGACTGCTCCAGCTTTACTTCCGGAACCTGAATTCTGCAGA

**S2 Fig. Experimentally determined anti-$GABA_A$ ß2/3 heavy chain sequence.**
