# Supplementary material for: Delivery of different genes into pre- and post-synaptic neocortical interneurons connected by GABAergic synapses
Source: PLoS One. 2019 May 24;14(5):e0217094. doi: 10.1371/journal.pone.0217094 (PMC6534327; doi:10.1371/journal.pone.0217094)
Supplement: S3 Fig — (PDF) [file pone.0217094.s003.pdf]

AAGCTTGGCGCGCCACCATGGCTGAAGCAAAGACCCACTGGCTTGGAGCAGCCCTGTCTCTTAT
CCCTTTAATTTTCCTCATCTCTGGGGCTGAAGCAGCTTCATTTCAGAGAAACCAGCTGCTTCAGAA
AGAACCAGACCTCAGGTTGGAAAATGTCCAAAAGTTTCCCAGTCCTGAAATGATCAGGGCTTTGG
AGTACATAGAAAACCTCCGACAACAAGCTCATAAGGGCTCGACAAGCGGTAGCGGCAAATCTAG
CGAAGGAAAGGGAGAAGTAAAGCTGGAGCAGTCAGGACCTGAGCTGGTGAAGCCTGGGGCCTC
AGTGAAGATTTCCTGCAAAGTTTCTGGCTACGAATTCAGTAGTTCTTGGATGAACTGGGTGAAAC
AGAGGCCTGGACAGGGTCTTGAGTGGATTGGACGGATTCATCCTGGAAATGGAGATGTTAAGTA
CAATGGGAAGTTCAAGGACAAGGCCACACTGACTGCAGACAAATCCTCCAGCACAGCCTACATG
GAGCTCAGCAGCCTGACCTCTGTGGACTCTGCGGTCTATTTCTGTGCAAAATGGGCCTGGGATG
AACACTGGGGCCAAGGCACCACTCTTACAGTCTCCTCAGGGGGTGGAGGAAGTGGTGGGGGCG
GATCTGGGGGAGGTGGCTCGGATATTGTGCTGACCCAGTCTCCTCTCACTTTGTCGGTTACCATT
GGACAACCAGCCTCCATCTCTTGCAAGTCAAGTCAGAGCCTCTTAGATAGTGATGGCAAGACATA
TTTGAATTGGTTGTTCCAGAGGCCAGGCCAGTCTCCAAAGCGCCTAATTTATCTGGTGTCTAAAC
TGGGCTCTGGAGTCCCTGACAGGTTCATTGGCAGTGGATCAGGGACAGATTTCACACTGAGAAT
CAGCAGAGTGGAGGCTGAGGATTTGGGAGTTTATTATTGCTGGCAAGGTACACATCTTCCTCGG
ACGTTTGGTGGAGGCACCAAGCTGGAAATCCAACGGGCTGATGCCAGCGCCGGAGGCGCCAGC
GGCGGAGCCAGCGCCGCAGGAGGCGCAAGTGCCGGCGCACATCACCATCACCATCACTAAAGA
TCTTTAATTAAGAATTC

S3 Fig. The DNA sequence for the synthetic peptide neurotransmitter dcv-secretogranin/anti-GABA$_A$ß2/3-HtoL/his-tag
