# Supplementary material for: Delivery of different genes into pre- and post-synaptic neocortical interneurons connected by GABAergic synapses
Source: PLoS One. 2019 May 24;14(5):e0217094. doi: 10.1371/journal.pone.0217094 (PMC6534327; doi:10.1371/journal.pone.0217094)
Supplement: S4 Fig — (PDF) [file pone.0217094.s004.pdf]

AAGCTTGGCGCGCCACCATGCCGAGATTCTGCTACAGTCGCTCAGGGGCCCTGTTGCTGGCCCT

CCTGCTTCAGACCTCCATAGATGTGTGGAGCTGGTGCCTGGAGAGCAGCCAGTGCCAGGACCTC

ACCACGGAGAGCAACCTGCTGGCTTGCATCCGGGCTTGCAAACTCGACCTCTCGCTGGAGACGC

CCGTGTTTCCTGGCAACGGAGATGAACAGCCCCTGACTGAAAACCCCCGGAAGTACGTCATGGG

TCACTTCCGCTGGGACCGCTTCGGCCCCAGGAACAGCAGCAGTGCTGGCAGCGCGGCGCAGAG

GCGTGCGGAGGAAGAGGCGGTGTGGGGAGATGGCAGTCCAGAGCCGAGTCCACGCGAGGGCA

AGCGCTCCTACTCCATGGGCTCGACAAGCGGTAGCGGCAAATCTAGCGAAGGAAAGGGAGATAT

TGTGCTGACCCAGTCTCCTCTCACTTTGTCGGTTACCATTGGACAACCAGCCTCCATCTCTTGCA

AGTCAAGTCAGAGCCTCTTAGATAGTGATGGCAAGACATATTTGAATTGGTTGTTCCAGAGGCCA

GGCCAGTCTCCAAAGCGCCTAATTTATCTGGTGTCTAAACTGGGCTCTGGAGTCCCTGACAGGTT

CATTGGCAGTGGATCAGGGACAGATTTCACACTGAGAATCAGCAGAGTGGAGGCTGAGGATTTG

GGAGTTTATTATTGCTGGCAAGGTACACATCTTCCTCGGACGTTTGGTGGAGGCACCAAGCTGG

AAATCCAACGGGCTGATGGGGGTGGAGGAAGTGGTGGGGGCGGATCTGGGGGAGGTGGCTCG

GAAGTAAAGCTGGAGCAGTCAGGACCTGAGCTGGTGAAGCCTGGGGCCTCAGTGAAGATTTCCT

GCAAAGTTTCTGGCTACGAATTCAGTAGTTCTTGGATGAACTGGGTGAAACAGAGGCCTGGACA

GGGTCTTGAGTGGATTGGACGGATTCATCCTGGAAATGGAGATGTTAAGTACAATGGGAAGTTCA

AGGACAAGGCCACACTGACTGCAGACAAATCCTCCAGCACAGCCTACATGGAGCTCAGCAGCCT

GACCTCTGTGGACTCTGCGGTCTATTTCTGTGCAAAATGGGCCTGGGATGAACACTGGGGCCAA

GGCACCACTCTTACAGTCTCCTCAGCCAGCGCCGGAGGCGCCAGCGGCGGAGCCAGCGCCGCA

GGAGGCGCAAGTGCCGGCGCACATCACCATCACCATCACTAAAGATCTTTAATTAAGAATTC

S4 Fig. The DNA sequence for the synthetic peptide neurotransmitter dcv-pomc/anti-GABA$_A$ß2/3-LtoH/his-tag.
