# Supplementary material for: Delivery of different genes into pre- and post-synaptic neocortical interneurons connected by GABAergic synapses
Source: PLoS One. 2019 May 24;14(5):e0217094. doi: 10.1371/journal.pone.0217094 (PMC6534327; doi:10.1371/journal.pone.0217094)
Supplement: S5 Fig — (A) Following gene transfer to connected neurons, labeling and counting of transduced axons that are proximal to, or distant from, a transduced dendrite. The experimental design and vectors followed Fig 3. The upper layers of POR cortex were examined. The photomicrograph shows a merge of the transduced axons (His tag-IR; Texas red-conjugated secondary antibody) and the transduced dendrites (GFP-IR; FITC-conjugated secondary antibody). Each transduced axon that is proximal to a transduced dendrite was labeled with a “+”; this image contains 79 of these axons. Inversely, each transduced axon that is distant from a transduced dendrite was labeled with a “-”; this image contains 12 of these axons. The targeting efficiency for the modest sample in this image is 79 / (79+12), or 87%. Multiple images, from multiple rats, were analyzed in this manner to generate the data in Table 2. (B) Following gene transfer to connected neurons, labeling and counting of the connected postsynaptic neurons that contain, or lack, parvalbumin. The experimental design and vectors followed Fig 8. The upper layers of POR cortex were examined. The photomicrograph shows a merge of the transduced axons (His tag-IR; fluorescein-conjugated secondary antibody), the transduced dendrites (GFP-IR; Alexa Fluor 633-conjugated secondary antibody), and parvalbumin-IR (TRITC-conjugated secondary antibody). The synapses that supported gene transfer to connected neurons were identified as in panel A and labeled with a “+”; this image contains 49 connected, transduced axons and dendrites. The postsynaptic neurons that also contain parvalbumin were scored by adding a “$”; this image contains 17 postsynaptic neurons that also contain parvalbumin. The percentage of postsynaptic neurons that also contain parvalbumin for the modest sample in this image is 17 / 49, or 35%. Multiple images, from multiple rats, were analyzed in this manner to generate the data in Table 3. Scale bar: 50 μm. (PDF) [file pone.0217094.s005.pdf]

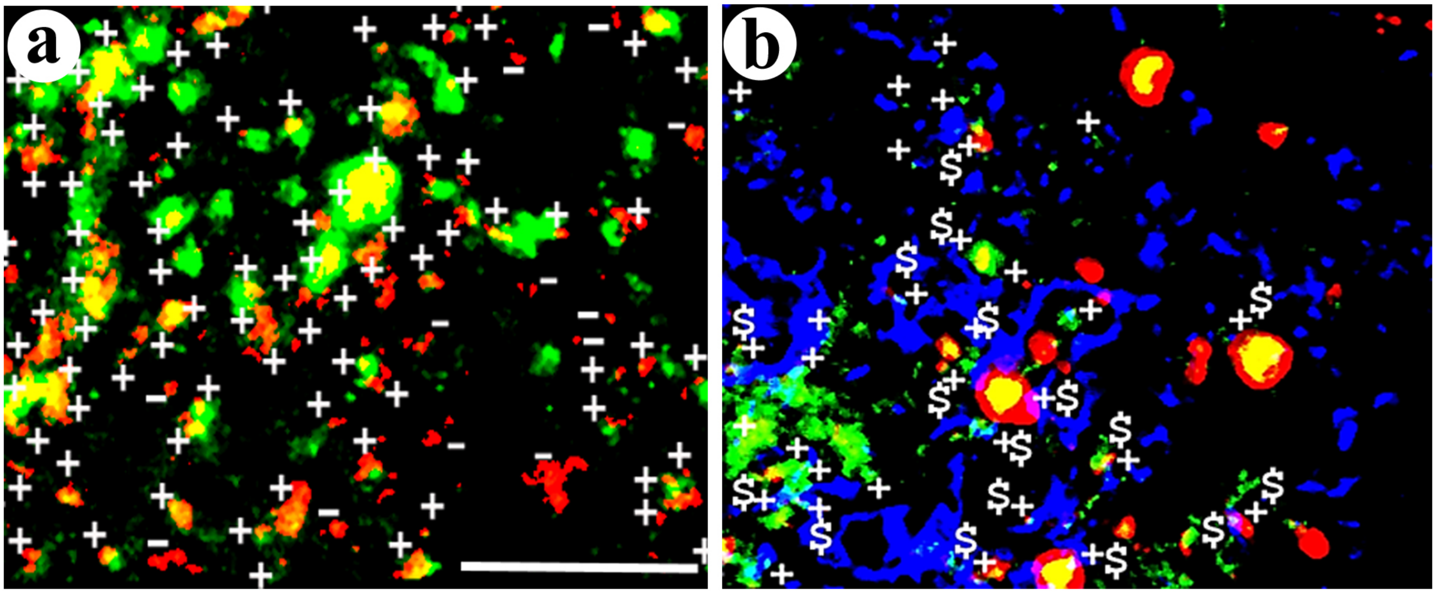

S5 Fig. Quantification of (A) the efficiency of gene transfer to connected neurons, or (B) the percentage of transduced postsynaptic neurons that also contain parvalbumin. (A) Following gene transfer to connected neurons, labeling and counting of transduced axons that are proximal to, or distant from, a transduced dendrite. The experimental design and vectors followed Figure 3. The upper layers of POR cortex were examined. The photomicrograph shows a merge of the transduced axons (His tag-IR; Texas red-conjugated secondary antibody) and the transduced dendrites (GFP-IR; FITC-conjugated secondary antibody). Each transduced axon that is proximal to a transduced dendrite was labeled with a "+"; this image contains 79 of these axons. Inversely, each transduced axon that is distant from a transduced dendrite was labeled with a "-"; this image contains 12 of these axons. The targeting efficiency for the modest sample in this image is 79 / (79+12), or 87 %. Multiple images, from multiple rats, were analyzed in this manner to generate the data in Table 2. (B) Following gene transfer to connected neurons, labeling and counting of the connected postsynaptic neurons that contain, or lack, parvalbumin. The experimental design and vectors followed Figure 8. The upper layers of POR cortex were examined. The photomicrograph shows a merge of the transduced axons (His tag-IR; fluorescein-conjugated secondary antibody), the transduced dendrites (GFP-IR; Alexa Fluor 633-conjugated secondary antibody), and parvalbumin-IR (TRITC-conjugated

secondary antibody). The synapses that supported gene transfer to connected neurons were identified as in panel A and labeled with a “+”; this image contains 49 connected, transduced axons and dendrites. The postsynaptic neurons that also contain parvalbumin were scored by adding a “$”; this image contains 17 postsynaptic neurons that also contain parvalbumin. The percentage of postsynaptic neurons that also contain parvalbumin for the modest sample in this image is 17 / 49, or 35 %. Multiple images, from multiple rats, were analyzed in this manner to generate the data in Table 3. Scale bar: 50 μm.
